# Characterization, mechanism of action and optimization of activity of a novel peptide-peptoid hybrid against bacterial pathogens involved in canine skin infections

**DOI:** 10.1038/s41598-019-39042-3

**Published:** 2019-03-06

**Authors:** Ines Greco, Agnete Plahn Emborg, Bimal Jana, Natalia Molchanova, Alberto Oddo, Peter Damborg, Luca Guardabassi, Paul R. Hansen

**Affiliations:** 1 Department of Drug Design and Pharmacology, Faculty of Health and Medical Sciences, University of Copenhagen, Universitetsparken 2, 2100 Copenhagen, Denmark; 2 Present Address: Department of Food Science, Faculty of Science, University of Copenhagen, Rolighedsvej 26, 1958 Frederiksberg, Denmark; 3 Present Address: Novo Nordisk, Brennum Park 1, 3400 Hilleroed, Denmark; 4 Department of Veterinary and Animal Sciences, Faculty of Health and Medical Sciences, University of Copenhagen, Stigbøjlen 4, 1870 Frederiksberg C, Denmark; 5 Present Address: Roskilde University, Department of Science and Environment, 4000 Roskilde, Denmark; 6 Present Address: Novo Nordisk A/S, Krogshøjvej 44, 2820 Bagsværd, Denmark; 7 Department of Pathobiology and Population Sciences, The Royal Veterinary College, Hawkshead Lane, North Mymms, AL9 7TA Hatfield, Herts, United Kingdom

**Keywords:** Antibiotics, Bacterial infection

## Abstract

Integumentary infections like pyoderma represent the main reason for antimicrobial prescription in dogs. *Staphylococcus pseudintermedius* and *Pseudomonas aeruginosa* are frequently identified in these infections, and both bacteria are challenging to combat due to resistance. To avoid use of important human antibiotics for treatment of animal infections there is a pressing need for novel narrow-spectrum antimicrobial agents in veterinary medicine. Herein, we characterize the *in vitro* activity of the novel peptide-peptoid hybrid **B1** against canine isolates of *S*. *pseudintermedius* and *P*. *aeruginosa*. **B1** showed potent minimum inhibitory concentrations (MICs) against canine *S*. *pseudintermedius* and *P*. *aeruginosa* isolates as well rapid killing kinetics. **B1** was found to disrupt the membrane integrity and affect cell-wall synthesis in methicillin-resistant *S*. *pseudintermedius* (MRSP). We generated 28 analogues of **B1**, showing comparable haemolysis and MICs against MRSP and *P*. *aeruginosa*. The most active analogues (**23**, **26**) and **B1** were tested against a collection of clinical isolates from canine, of which only **B1** showed potent activity. Our best compound **26**, displayed activity against *P*. *aeruginosa* and *S*. *pseudintermedius*, but not the closely related *S*. *aureus*. This work shows that design of target-specific veterinary antimicrobial agents is possible, even species within a genus, and deserves further exploration.

## Introduction

*Staphylococcus pseudintermedius* is a commensal bacterium colonizing dog skin and mucosal sites^1^, and it is the predominant cause of canine pyoderma and otitis externa^2^. These common infections represent the main reason for antimicrobial prescription in dogs^3^. Over the last decade, methicillin-resistant *S*. *pseudintermedius* (MRSP) has been reported worldwide^4^, including sporadic infections in humans in contact with dogs^5,6^. *Pseudomonas aeruginosa* is another pathogen frequently involved in canine integumentary infections, in particular otitis externa^7^. *P*. *aeruginosa* is resistant to most antibiotics used in veterinary medicine. The presence of this pathogen and the increasing frequency of multidrug-resistant MRSP^8^, make treatment of dogs with integumentary infections difficult or even impossible in some cases^9^. In light of the few treatment options available against these pathogens, new therapeutic agents are needed, preferably drugs restricted to veterinary use and with a narrow spectrum^10^. This would limit their impact on the commensal microbiota^11^.

In recent years, antimicrobial peptides (AMPs) have attracted considerable interest as alternative anti-infectives^12^. AMPs are present in all multicellular organisms as part of their innate immune systems^13^. They show selective toxicity towards bacteria, rapid killing, broad-spectrum antimicrobial activity, and are active at micromolar concentrations or lower^14^. Furthermore, they possess immunomodulatory properties such as leukocyte recruitment and suppression of harmful inflammation^15^. Most AMPs exhibit their antimicrobial activity by disrupting the bacterial cell membrane; however, intracellular targets have also been reported^16^.

The main drawbacks of AMPs as therapeutics are toxicity and susceptibility to proteases^17^. Traditionally, these problems are overcome by chemical modification, such as cyclization or design of peptidomimetics, which are stable to proteases^18^. We and others have previously investigated antimicrobial *N*-alkylglycines (peptoids)^19^, β-peptoids (N-alkyl-β-alanine oligomers)^20^, β-peptides^21^, lysine-based α-peptide/β-peptoids^22^, and α/γ *N*-Acylated-*N*-aminoethylpeptides (AApeptides)^23^. Some studies have reported activity of peptides and peptidomimetics against veterinary pathogens^24^. However, only a few of them aimed at the design and optimization of AMPs with activity against *S*. *pseudintermedius*^25–28^ and canine strains of *P*. *aeruginosa*^29^.

**Figure 1:**
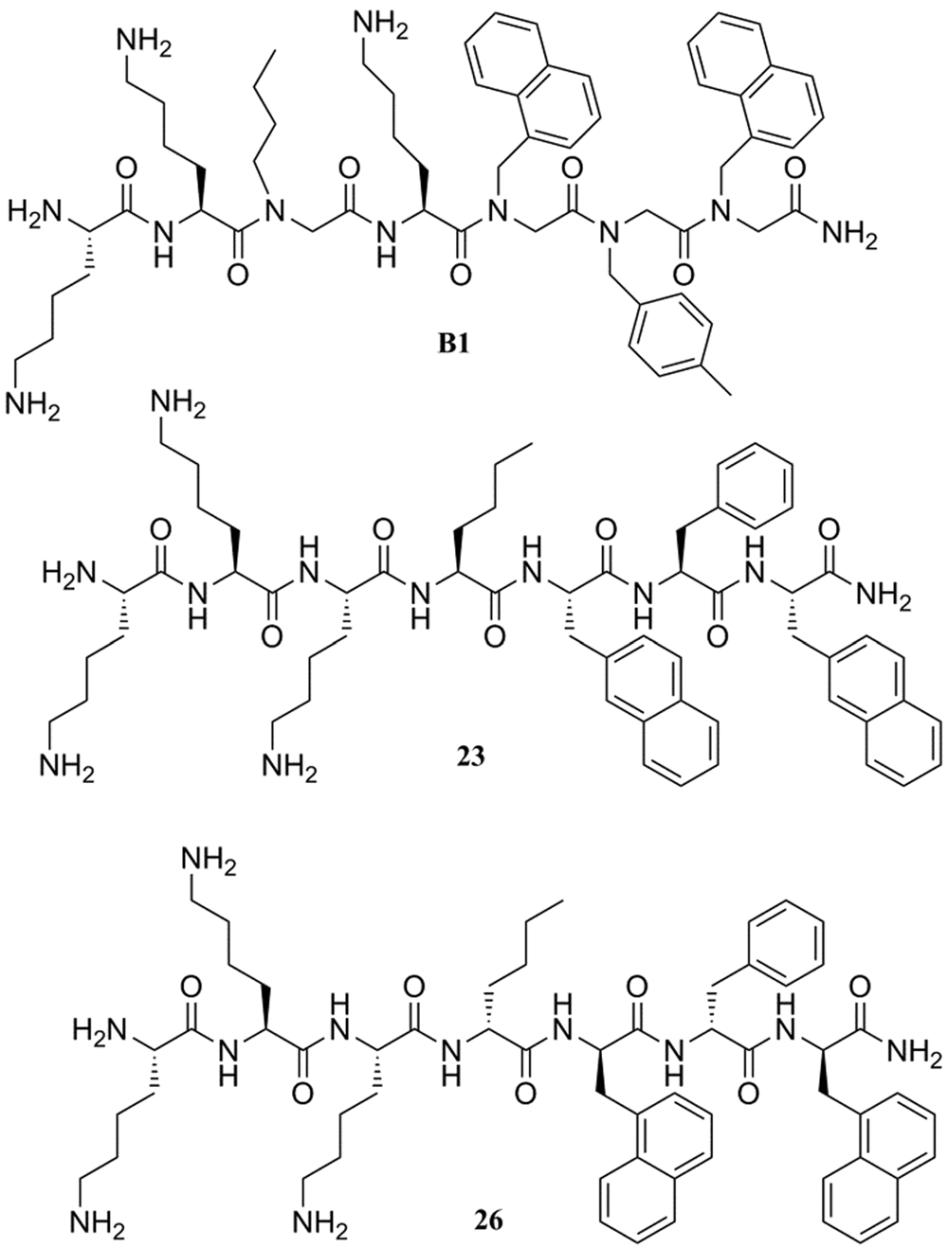
Lead compound **B1** and optimized structures **23** and **26**.

The peptide-peptoid hybrid **B1** (Fig. 1) has been previously identified and described as active against one clinical isolate of *S*. *pseudintermedius* and *P*. *aeruginosa*, as well as resistant to proteolytic degradation in conditions resembling *in vivo* metabolism^30^. The aim of the present study was to investigate the antimicrobial activity of **B1** against a large collection of *S*. *pseudintermedius* and *P*. *aeruginosa* isolates from canine infections, determine time-kill kinetics and probe the mechanism of action against *S*. *pseudintermedius*. Furthermore, we designed and tested 28 analogues of **B1** and selected the peptides **23** and **26** (Fig. 1) for their improved selectivity against *S*. *pseudintermedius* compared to **B1** while retaining comparable activity against *P*. *aeruginosa*. Next, we aimed to get insight into the antibacterial activity of **B1**, **23** and **26** against other bacterial species causing infections in dogs. Finally, we closely investigated the selectivity of compounds **23** and **26** against *S*. *pseudintermedius* relative to *S*. *aureus*. This study demonstrates that design of peptide-based antimicrobials which target specific veterinary bacterial species is possible.

## Results

### Antimicrobial activity and killing kinetics

Compound **B1** was identified from a combinatorial library. Further evaluation of **B1’s** antimicrobial activity against a panel of 57*S*. *pseudintermedius* isolates from canine infections revealed consistently low MICs (2–4 µg/mL), irrespective of methicillin resistance (Fig. S5, Supplementary Information). Similar MICs (4–8 µg/mL) were observed for the three *S*. *aureus* isolates tested (data not shown). Furthermore, MICs of **B1** against a panel of 50*P*. *aeruginosa* isolates ranged from 8–16 µg/mL.

**Figure 2:**
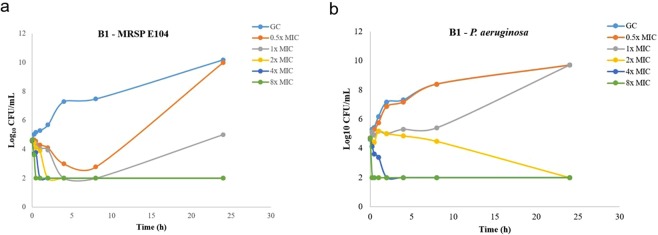
Time kill kinetics for **B1**. Time kill kinetics for **B1** against (**a**) MRSP E104 and (**a**) *P*. *aeruginosa* 26314. Time kill assays were performed in triplicate and presented as the average of three different samplings.

The time-kill kinetic assay showed complete killing of the tested MRSP strain E104 (MIC = 8–16 µg/mL) in 2 h at 2 × MIC, and in 1 h at 4 × MIC, revealing a rapid concentration-dependent effect (Fig. 2a). A similar but slightly inferior effect was detected against the clinical strain *P*. *aeruginosa* 26314, which was killed at 4x MIC in 2 h and at 2x MIC in 24 h (Fig. 2b). Lower concentrations (1x MIC) of **B1** did not eliminate the *Pseudomonas* strain but resulted in delayed re-growth (Fig. 2b).

### Mode of action of B1 against *S*. *pseudintermedius* E104

The mode of action of **B1** was investigated by studying the effect of sub-inhibitory concentrations of **B1** on membrane potential and macromolecule synthesis rate of the MRSP strain E104. The well-characterized antimicrobial nisin was used as a control. Growth curve analysis of **B1** at 3 µg/ml showed cell lysis, as indicated by a decrease of OD over time (Fig. S6 Supplemental Material). However, at lower concentrations, **B1** resulted in only minor growth inhibition. This is in contrast with nisin, which in addition to cell lysis at MIC concentration (5 µg/ml), significantly retarded growth, also at sub-inhibitory concentrations.

**Figure 3:**
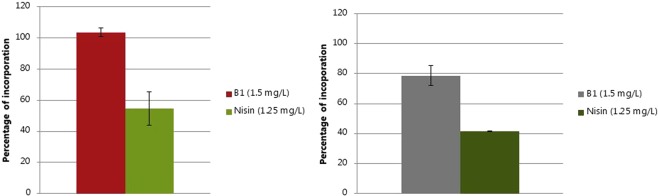
Effects of B1 and nisin on DNA (**a**) and cell wall (**b**) synthesis as measured by macromolecule biosynthesis analysis. Percentages of DNA and cell wall precursors incorporation with respect to unexposed control are presented as average values of two individual measurements.

In order to determine if the lysis of MRSP E104 by **B1** was due to the inhibition of cell wall biosynthesis, the synthesis rate of cell wall macromolecules was studied at sub-inhibitory concentrations (1.5 µg/mL). In addition, since cationic AMPs have been reported to interact with DNA due to their positive charge and hydrophobicity, the DNA replication was also measured. **B1** resulted in a 20% reduction in cell wall synthesis without affecting DNA replication (Fig. 3). Exposure to nisin resulted in 50% inhibition of DNA synthesis (Fig. 3a) and 60% inhibition of cell wall synthesis (Fig. 3b)

**Figure 4:**
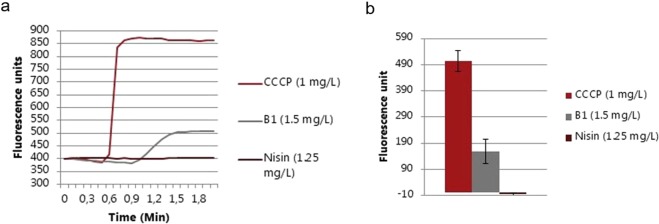
Effects of B1, Carbonyl cyanide *m*-chlorophenylhydrazone (CCCP) or nisin on DiSC3(5) (3,3′-Dipropylthiadicarbocyanine Iodide) fluorescence. Effects of B1, Carbonyl cyanide *m*-chlorophenylhydrazone (CCCP) or nisin on DiSC3(5) (3,3′-Dipropylthiadicarbocyanine Iodide) fluorescence plotted by emission spectra (**a**) and increment of fluorescence after exposure (**b**).

**Figure 5:**
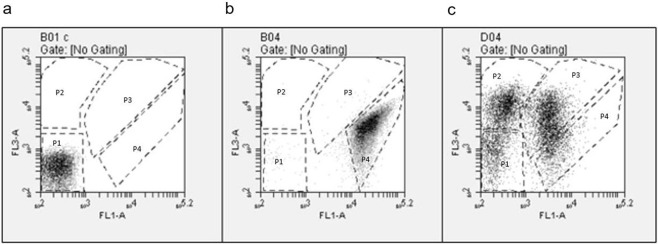
Flow cytometry analysis of MRSP E104 exposed to B1. Four different conditions of cells are represented by boxes: P1 (unstained), P2 (dead cells), P3 (injured cells) and P4 (live cells). FL1 and FL3 axis represent green (thiazole orange, TO) and red fluorescence (propidium iodide, PI), respectively. (**a**) is a dotted plot of unstained cells, (**b**,**c**) are TO/PI stained unexposed and **B1** exposed cells, respectively.

To test the immediate effect of a sub-inhibitory concentration of **B1** (1.5 µg/mL) on membrane potential, proton motive force (PMF) was measured using DiSC_3_(5) (3,3’-Dipropylthiadicarbocyanine Iodide), a fluorescent probe that concentrates in energized membranes and is released in the environment surrounding a cell upon membrane depolarization, thus increasing the intensity of its emission. As expected, no fluorescence increment was observed after nisin treatment (Fig. 4a,b). On the contrary, **B1** caused significant membrane depolarization, as indicated by an increased DiSC_3_(5) fluorescence emission (Fig. 5a,b). Furthermore, the energy dissipation effect of **B1** on the cell at the lysis concentration (1x MIC) was studied by flow cytometry analysis. Cell death (Fig. 5c, P2) and injury (P3) upon **B1** exposure was indicated by the high ratio of propidium iodide (PI) staining relative to thiazole orange (TO) staining, whereas unexposed control cells were mainly stained with TO (Fig. 5b). Taken together, our studies indicated that **B1** causes membrane depolarization and affects cell-wall synthesis but not DNA-synthesis.

### Analogues of B1

After characterizing **B1** as a lead, we proceeded to generate an ensemble of 28 analogues (Table S1, Supplementary Information) in order to develop compounds with specific activity against *S*. *pseudintermedius* and *P*. *aeruginosa*, paired with low activity against methicillin-susceptible *Staphylococcus aureus* (MSSA). Compounds **2–29** are analogues of **B1** (Fig. 1A). Compounds **2**–**5** (Table 1) contain L-Lys and peptoid residues. In compound **2**, residue 6 (*N*-4-methylbenzylglycine) was replaced with *N*-benzylglycine resembling Phe; in compound **3**, residue 4, *N*-butylglycine, was switched with residue 5, Lys, to generate a hydrophobic and cationic cluster. Compounds **4** and **5** are the reversed compounds of **2** and **3**.

**Table 1:** Sequence, Minimum Inhibitory Concentration in µg/mL and Haemolysis (µM) for compound B1 and 28 analogs.

ID	Sequence^a^	Minimum Inhibitory Concentration (µg/ml)			Haemolysis (µM)		
		MRSP C22963	MSSA (ATCC 29213)	*P*. *aeruginosa* (26314)	EC_10_	EC_50_	%H 150(µM)
**B1**	Lys-Lys-(*N*Bu)Gly-Lys-(*N*1-Nal)Gly-(*N*4-MeBn)Gly-(*N*1-Nal)Gly	2–4^b^	8–16	8–16	64	230	32
**2**	Lys-Lys-(*N*Bu)Gly-Lys-(*N*1-Nal)Gly-(*N*Phe)Gly-(*N*1-Nal)Gly	4	8–16	32	—	—	<8
**3**	Lys-Lys-Lys-(*N*Bu)Gly-(*N*1-Nal)Gly-(*N*Phe)Gly-(*N*1-Nal)Gly	4	16	32	—	—	<8
**4**	(*N*1-Nal)Gly-(*N*Phe)Gly-(*N*1-Nal)Gly-(*N*Bu)Gly-Lys-Lys-Lys	8	16	32	54	—	24
**5**	(*N*1-Nal)Gly-(*N*Phe)Gly-(*N*1-Nal)Gly-Lys-(*N*Bu)Gly-Lys-Lys	8	16	32	—	—	<8
**6**	Lys-Lys-Leu-Lys-(1-Nal)Ala-Phe-(1-Nal)Ala	8	32	8	5	58	96
**7**	Lys-Lys-Lys-Leu-(1-Nal)Ala-Phe-(1-Nal)Ala	8	16	16	8	140	55
**8**	Lys-Lys-Leu-Lys-(2-Nal)Ala-Phe-(2-Nal)Ala	8	16	32	56	—	34
**9**	Lys-Lys-Lys-Leu-(2-Nal)Ala-Phe-(2-Nal)Ala	8	16	16	46	—	39
**10**	(1-Nal)Ala-Phe-(1-Nal)Ala-Lys-Leu-Lys-Lys	8–16	16–32	64	—	—	<8
**11**	(2-Nal)Ala-Phe -(2-Nal)Ala-Lys-Leu-Lys-Lys	4–8	16	64	—	—	<8
**12**	(1-Nal)Ala-Phe-(1-Nal)Ala-Leu-Lys-Lys-Lys	16	32	>64	—	—	<8
**13**	(2-Nal)Ala-Phe-(2-Nal)Ala-Leu-Lys-Lys-Lys	8	32	>64	110	—	15
**14**	Lys-Lys-leu-Lys-(1-Nal)ala-phe-(1-Nal)ala	4–8	16	16–32	28	128	58
**15**	Lys-Lys-Lys-leu-(1-Nal)ala-phe-(1-Nal)ala	2	8	8	8	40	88
**16**	Lys-Lys-Lys-leu-(2-Nal)ala-phe-(2-Nal)ala	2–4	8–16	32	18	118	60
**17**	Lys-Lys-leu-Lys-(2-Nal)ala-phe-(2-Nal)ala	8	16	32	8	—	40
**18**	(2-Nal)ala-phe-(2-Nal)ala-Lys-leu-Lys-Lys	16	32–64	>64	—	—	<8
**19**	(2-Nal)ala-phe-(2-Nal)ala-leu-Lys-Lys-Lys	16	32	>64	—	—	<8
**20**	(1-Nal)ala-phe-(1-Nal)ala-Lys-leu-Lys-Lys	16	32–64	>64	—	—	<8
**21**	(1-Nal)ala-phe-(1-Nal)ala-leu-Lys-Lys-Lys	16	32	32	—	—	<8
**22**	Lys-Lys-Lys-Leu-(2-Nal)Ala-Tyr-(2-Nal)Ala	16	>64	>64	117	—	14
**23**	Lys-Lys-Lys-Nle-(2-Nal)Ala-Phe-(2-Nal)Ala	2–4	32–64	8	14	104	59
**24**	Lys-Lys-Lys-Nle-(1-Nal)Ala-Phe-(1-Nal)Ala	16–32	>64	16	3	41	60
**25**	Lys-Lys-Lys-leu-(1-Nal)ala-tyr-(1-Nal)ala	2–4	32–64	8	38	138	53
**26**	Lys-Lys-Lys-nle-(1-Nal)ala-phe-(1-Nal)ala	4–8	>64	8	—	63	56
**27**	Lys-Lys-Lys-Nle-(2-Nal)Ala-Tyr-(2-Nal)Ala	2–4	64	64	3	56	54
**28**	Lys-Lys-Lys-leu-(2-Nal)ala-tyr-(2-Nal)ala	2–4	32	32	6	50	64
**29**	*NLys*-*NLys*-*NLys*-Leu-(2-Nal)Ala-Phe-(2-Nal)Ala	4–8	16–32	32	9	—	46

^a^All compounds were synthesized as peptide amides and isolated as TFA salts. ^b^MRSP strain E104 (MIC = 8–16 µg/mL).

Compounds **6**–**13** contain L-lysine and L-amino acids (Leu, Phe, 1-Nal, 2-Nal) instead of the peptoid residues N-butylglycine, N-1-naphthylmethylglycine and *N*-4-methylbenzylglycine, respectively. In compound **6**, peptoid residues have been replaced with the corresponding amino acids and in **7**, residue Leu^4^ and residue Lys^5^ have been switched to obtain a hydrophobic and cationic cluster. Compounds **8** and **9** differ from **6** and **7** for the presence of 2- instead of 1-Nal. Compounds **10**–**13** are the reverse of **6**–**9**. Compounds **14**–**21** are analogues of **6**–**13** in which L-Lys residues have been retained and Leu, 1-Nal, 2-Nal and Phe have been replaced by the corresponding D-stereoisomers.

Based on the data for **1**–**21**, we synthesized a second set of compounds (**22–29**) maintaining three L-Lys at the N-terminus except for **29**. Compounds **22–23** are analogues of **9** in which Phe^6^ and Leu^4^ were replaced by Nle and Tyr, respectively. Compound **23** is an analogue of **7** in which Leu^4^ was replaced with Nle. Compounds **25** and **26** are derived from **15**, in which D-Phe^6^ and D-Leu^4^ have been replaced by D-Nle and D-Tyr, respectively. Compounds **27** and **28** contain combinations of substitutions introduced in compounds **22–26**. Finally, compound **29** is an analogue of **9** in which Lys residues have been replaced by N-(4-aminobutyl)glycine.

MICs of the analogues were determined against *S*. *pseudintermedius* C22963, MSSA (ATCC 29213), and *P*. *aeruginosa* 26314. The sequence of each analog, the MICs and hemolysis data against red blood cells are reported in Table 1.

Compounds **2–21** were generally less active than **B1** with MICs of 2–16 µg/mL against *S*. *pseudintermedius* C22963, 8–64 µg/mL against *S*. *aureus*, and 8- >64 µg/mL against *P*. *aeruginosa* 26314. Hemolysis at 150 µM for compounds **2–17** ranged from <8–96% while the reverse sequences **18–21** were not hemolytic.

To further improve selectivity and hemolysis, we synthesized a second set of compounds (**22–29**) maintaining three L-Lys at the N-terminus except for **29**. Five of the compounds featuring three Lys (or Lys-like residues, Fig. 1) at the N-terminus (**23**, **25**, **26**, **27** and **28**) showed 16-fold better activity against the MRSP strain (MIC 2–4 µg/mL) than against the MSSA strain (MIC 32- >64 µg/mL). Furthermore, they showed moderate activity against MRSA USA300 strain FPR3757 (32- >64 µg/mL) (data not shown). In addition, compound **23**, **25** and **26** retained the activity level of **B1** against *P*. *aeruginosa* (8 µg/mL). The haemolytic values of compounds **22–29** ranged from 46 to 64% at 150 µM except for compound **22** (14%). The two most promising analogues (**23** and **26**) were selected for further studies due to (i) the low MICs observed in MRSP and *P*. *aeruginosa*, (ii) the relatively higher antimicrobial effect against *S*. *pseudintermedius* compared to *S*. *aureus*, and (iii) a moderate to low-level of hemolysis EC_50_ of 104 µM and 63 µM, respectively (Table 1).

### Time-kill experiments of 23 and 26

**Figure 6:**
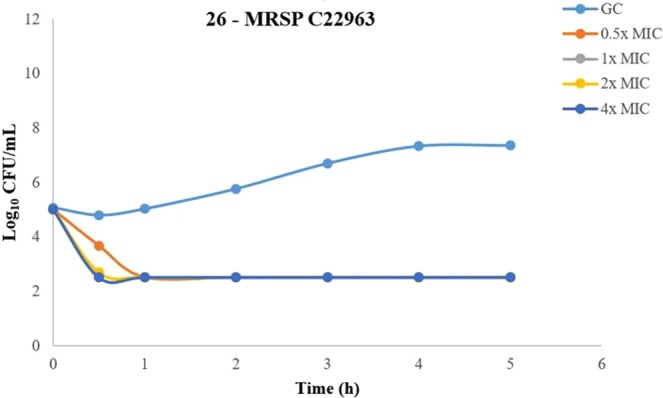
Time kill curve of compound 26 against MRSP C22963. Time kill curve of compound **26** against MRSP C22963 at four different concentrations. Time kill assays were performed in triplicate and presented as the average of three different samplings.

**Figure 7:**
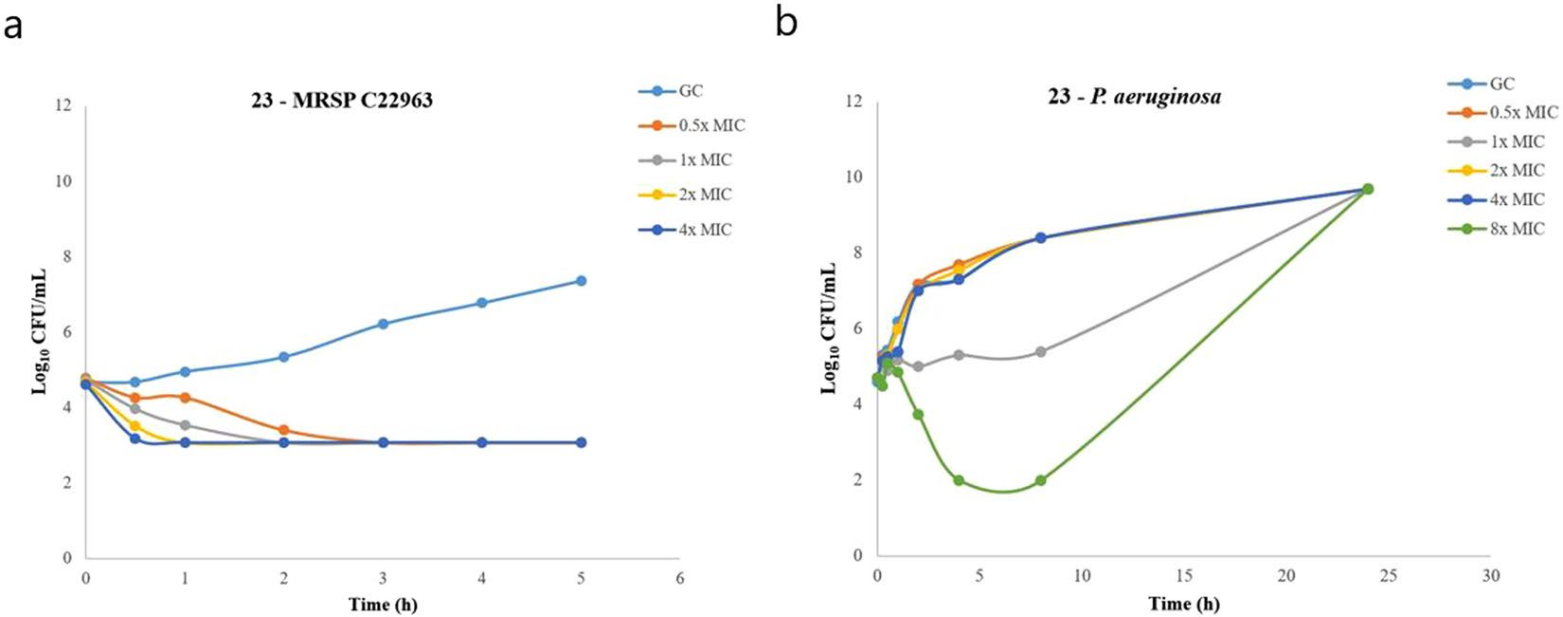
Time kill kinetics for 23. Time kill kinetics for 23 against (**a**) MRSP C22963 and (**a**) *P*. *aeruginosa* 26314. Time kill assays were performed in triplicate and presented as the average of three different samplings.

Time-kill experiments showed that **26** (Fig. 6). was superior to both **B1** and **23** (Fig. 7a) and displayed complete killing of MRSP C22963 within 1 h at only 0.5x MIC Similarly to **B1**, these two analogues exhibited complete and concentration-dependent killing of the MRSP strain within 2 h at all tested concentrations at or above the MIC. Surprisingly, **23** was not able to completely kill *P*. *aeruginosa*, even at the highest tested concentration (8x MIC) (Fig. 7b). We also intended to do time-kill curves for **26** against *P*. *aeruginosa* (MIC = 16 µg/mL). To our disappointment, **26** precipitated after 15 min in the media, and it was not possible to get reproducible results.

### Antimicrobial activity of B1, 23 and 26 against a selection of canine pathogens

In order to get further insight into the antimicrobial spectrum of **B1**, **23** and **26**, we tested MICs against a collection of clinical isolates representing other canine pathogens (*Corynebacterium auriscanis*, *Enterococcus faecalis*, *Enterococcus faecium*, *Streptococcus canis*, *Acinetobacter baumannii*, *Escherichia coli*, *Klebsiella pneumonia*, *Pasteurella canis*, *Proteus mirabilis*) (Table 2). All three compounds showed activity against Gram-positive bacteria with MICs ranging between 2–16 µg/mL, except for *Enterococcus* (>64 mg/mL). However, **B1**, **23** and **26** were substantially less active against the Gram-negative bacteria with most of the MICs in the range of 32- >64 µg/mL, *P*. *aeruginosa and Pasteurella canis* being the only exceptions. For the latter species, the effect varied consistently between **B1**, **23** and **26** (8 µg/mL, >64 µg/mL and >64 µg/mL, respectively).

**Table 2:** Antimicrobial activity of B1, 23 and 26 against a collection of clinical isolates from canine (µg/mL).

Bacteria	B1	23	26
*Acinetobacter baumannii*, 27065, 16 D1, dog, wound, 2010	64	64	>64
*Corynebacterium auriscanis*, 31551, 54 C6, dog, ear, 2013	4	8	32
*E*. *coli*, 30235, 23 A6, dog, wound, 2012	64	32	32
*Enterococcus faecalis*, 27404, 17 C7, dog, wound, 2011	64	>64	64
*Enterococcus faecium*, 30951, 24 C1, dog, ear, 2013	16	64	>64
*Klebsiella pneumoniae*, 26233, 11 H5, dog, wound, 2010	>64	>64	>64
*Pasteurella canis*, 31096, 24 C8, dog, skin, 2013	8	>64	>64
*Proteus mirabilis*, 25178, 9 A4, dog, ear, 2009	>64	>64	>64
*Pseudomonas aeruginosa (26314*, *12 C5*, *dog*, *urine*, *2010)*.	16	64	>64
*Streptococcus canis*, 26740-1, 14 H1, dog, ear, 2010	4	16	>64
*Staphylococcus aureus*, 27266, 16 G9, dog, skin, 2010	8	32	64
*Staphylococcus pseudintermedius (22963*, *3 B9*, *dog*, *2007)*.	2	4	4

### Selectivity of 23 and 26 between *S*. *pseudintermedius* and *S*. *aureus*

Finally, we tested the selectivity of 23 and 26 against clinical isolates of *S*. *pseudintermedius* (n = 10) vs *S*. *aureus* (n = 10) (Table S7, supplemental material). We found that the two compounds were generally more active against *S*. *pseudintermedius* (2–16 µg/mL) than *S*. *aureus* (32- >64 µg/mL).

## Discussion

The aims of this study were to (i) characterize the antimicrobial activity of the novel peptide-peptoid hybrid **B1** against a large collection of two common dog integumentary pathogens *S*. *pseudintermedius* and *P*. *aeruginosa*; (ii) probe the mode of action against *S*. *pseudintermedius* E104*;* (iii) conduct a structure-activity study of **B1** involving 28 analogues and identify compounds with potent activity against representative strains of *S*. *pseudintermedius* and *P*. *aeruginosa*, paired with weak activity against *S*.*aureus*; (iv) test the most promising of these analogues (**23** and **26**) as well as **B1** against a broad panel of other canine pathogens; (v) test for selectivity within the *Staphylococcus* genus between *S*. *pseudintermedius* and *S*. *aureus*.

**B1** showed low MICs against 50 canine *P*. *aeruginosa* (8–16 µg/mL), 57*S*. *pseudintermedius* (2–4 µg/mL). Killing kinetics showed that B1 kills MRSP and *P*. *aeruginosa* in less than 30 min at 8 x MIC. Our MIC and time-kill data for MRSP and *P*. *aeruginosa* are comparable with previous literature reports. Mohamed *et al*. designed and tested synthetic peptides (8 to 16 amino acids) against MSSP and MRSP. The most effective peptides displayed a MIC_50_ and MIC_90_ of 1 and 2 µM, respectively^25^. Molchanova *et al*.^22^ reported 22 different α-peptide/β-peptoid hybrids containing cationic and hydrophobic residues in a 1:1 ratio that were active against MRSP (2–8 µg/mL) as well as other relevant Gram-positive and Gram-negative bacteria. The same authors also identified fluorinated antimicrobial lysine-based peptidomimetics with activity against methicillin-resistant *S*. *pseudintermedius*^26^. Finally, Cabassi and coworkers identified a peptide (AMP2041) with activity against human and animal multidrug resistant *P*. *aeruginosa* isolates^31^, including strains of canine origin. In a parallel study to the present, we have characterized the *in vitro* pharmacokinetic properties of **B1**, including hemolytic activity and stability to proteases. The measured hemolytic activity of **B1** was 32% at 150 µM and the compound showed only 38% degradation after 24 hours exposure to the mix of protease of bacterial origin Pronase. Furthermore, **B1** was suitable for topical delivery from cream formulation and showed no skin penetration after administration^30^. These data, combined with our results from the present study, suggest that **B1** may be suitable as antimicrobial for topical treatment of canine superficial pyoderma.

To investigate the mode of action of **B1**, we used the well-characterized clinical MRSP ST71 strain E104 (MIC = 8–16 µg/mL), which is resistant to β-lactams, ciprofloxacin, clindamycin, doxycycline, and trimethoprim/sulfamethoxazole^8^. We found that the primary mode of action of **B1** is on the bacterial membrane and secondarily on cell wall synthesis. However, **B1** has no effect on DNA replication. Membrane activity was supported by a reduction of the initial OD in the growth curve study at 3 µg/mL (Fig. S6, Supplementary information), indicating cell lysis. Furthermore, **B1** caused significant membrane depolarization as seen by an increased DiSC_3_(5) fluorescence emission (Fig. 4a,b). We obtained further support for the membrane permeabilization by the rapid cell death observed in the flow cytometry assay (Fig. 5).

The primary mode of action of **B1** is in agreement with the classical membrane-targeting mechanism reported for a number of antimicrobial peptides, e.g. magainin II^32^ and Cecropin B^33^. Besides membrane disruption, AMPs may have intracellular targets as reviewed recently^34^. These include protein^35^, DNA^36^, and cell-wall synthesis^37^. Here, **B1** reduced cell wall synthesis by 20% (Fig. 3b). A few antimicrobial peptides such as plectasin have been reported to inhibit cell wall synthesis in Gram-positive bacteria. Plectasin targets the bacterial cell wall precursor lipid II as determined by advanced NMR^38^.

Our finding that **B1** does not affect DNA synthesis (Fig. 3a), is in agreement with literature reports that most AMPs do not have this target. However, some exceptions are known, e.g. indolicidin^39^. Furthermore, LP5, a compound similar to **B1**, has been reported to inhibit DNA replication and induce SOS response in *S*. *aureus*^40^. This may be due to a difference in net charge +4 (**B1**) and +6 (**LP5**), respectively.

In the mechanism of action study of **B1**, we used the well-characterized antimicrobial agent nisin as control. This antimicrobial compound used in food presevation interacts with the peptidoglycan precursor molecule lipid II, which leads to membrane depolarization and bacterial cell death^41^. The mechanism of nisin has been studied using a plethora of different techniques^42^^,^^43^.In our study, nisin had an inhibitory effect on MRSP growth at sub-lysis concentrations (Fig. S6 Supplementary Information). Simultaneous cell lysis and growth inhibition effects by nisin may be linked to the dual mode of action of this AMP, which encompasses both membrane pore formation and cell wall synthesis inhibition. Exposure to nisin resulted in 60% inhibition of cell wall synthesis, and 50% inhibition of DNA synthesis (Fig. 3a,b). We did not investigate the mechanism of **B1** on *P*. *aeruginosa*, but a few previous reports on the AMP killing mode of action of *P*. *aeruginosa* exist: using fluorescence microscopy and field emission scanning electron microscopy, Memariani *et al*. found that the 14-mer AMP PV3, kills bacteria by disrupting the cell membrane^44^. Furthermore, Scocchi and coworkers investigated the mechanism of killing against *P*. *aeruginosa* strain PAO1 and additional three isolates and observed that Bac7 (1–35) inactivated the target cells by disrupting their cellular membranes^45^.

We synthesized 28 analogues of **B1** by (i) altering the peptoid residues position in the sequence; (ii) substituting peptoid residues for L-amino acids; (iii) modifying the chirality of the amino acid components and/or altering their position in the sequence. Like **B1** and most AMPs, these analogues are cationic and hydrophobic. The rationale for their design is further discussed in results section. Besides *S*. *pseudintermedius* and *P*. *aeruginosa*, all the analogues were also tested against MSSA (ATCC 29212). Our two best compounds, **23** and **26**, showed slightly higher MICs (Table 1) against *S*. *pseudintermedius* (2–4 µg/mL and 4–8 µg/mL respectively) and *P*. *aeruginosa* (8 µg/mL) compared to **B1** and considerably worse activity against MSSA (32–64 µg/mL, >64 µg/mL), respectively).

Hemolysis at 150 µM is a commonly used parameter in the literature for comparison of antimicrobial peptides or peptidomimetics^46^. The hemolytic activity of **23** and **26** was 59% and 56% at 150 µM, respectively. A variation of the therapeutic index, selectivity index, SI, is often used in the field and is defined as the ratio between the concentration leading to 50% lysis of human erythrocytes and the minimum concentration inhibiting bacterial growth SI = (HC_50_/MIC) for the bacterium being considered^47^. The selectivity indices for MRSP C22963 are: **B1** (82), **23** (37), **26** (11). Typically, selectivity indices are below 100, although higher values have been reported^48^.

The SI values are not a major issue for drugs intended for topical use, since a highly hemolytic compound like the steroid antibiotic fusidic acid is being used for against human skin disorders and canine pathogenic staphylococci^49^. Compound **23** showed slower killing kinetics against MRSP C22963 and *P*. *aeruginosa* than **B1**, and was not able to eliminate *P*. *aeruginosa* but resulted in delayed re-growth. Notably, compound **26** was able to kill MRSP C22963 within 1 h at only 0.5x MIC, which was faster than both **B1** and **23** and fully comparable with literature reports^25^.

We tested the activity of **B1** and the analogues **23** and **26** against a broader range of canine pathogens. They displayed antimicrobial activity (Table 2) against a collection of Gram-positive clinical isolates (2- >64 µg/mL) and less against the Gram-negative isolates (8- >64 µg/mL). Generally, **B1** proved a lot more active than **23** and especially **26** (Table 2). In addition to *S*. *pseudintermedius*, *S*. *aureus* and *P*. *aeruginosa*, we observed increased activity for **B1** (4–16 µg/mL) against other bacteria such as: *Corynebacterium auriscanis* associated with canine otitis externa^50^, *Streptococcus canis* which causes respiratory, cutaneous, genital and urinary infections in various animal species^51^, *Pasteurella canis* which is a well-known major pathogen of infections caused by dog bites^52^, and *Enterococcus faecium* which is an important nosocomial pathogen^53^. However, we observed no significant activity against bacterial isolates belonging to the species *Acinetobacter baumannii*, *Escherichia coli*, *Enterococcus faecilis and Klebsiella pneumoniae*.

Having established the antimicrobial spectrum of **23** and **26**, we tested the selectivity of **23** and **26** against clinical isolates of *S*. *pseudintermedius* (n = 10) vs *S*. *aureus* (n = 10) (Table S7, supplementary information). We found that the two compounds were generally more active against *S*. *pseudintermedius* (2- >16 µg/ml) than *S*. *aureus* (32- >64 µg/mL), especially compound **26**. AMPs with enhanced selectivity against a target bacterial genus have been previously reported^54^ and selectivity within a genus has been observed in few other studies^55^. For example, Guo *et al*. published a peptide, C16G2, which is able of killing *S*. *mutans* selectively but not closely related streptococcal species. Our finding is significant, as we demonstrated that short amphipathic peptidomimetics can maintain activity against *S*. *pseudintermedius*, even when their efficacy against *S*. *aureus* decreases multiple folds. This suggests that the former might be more susceptible to membrane-active agents, or susceptible to a wider range of agents. These observations should aid the design of novel therapeutics for the treatment of *S*. *pseudintermedius* infections in animals, for which **B1**, **23** and **26** pose as promising lead candidates. The underlying reason for the higher activity of compounds **23** and **26** against *S*. *pseudintermedius* over *S*. *aureus* is unknown, but might be related to peptides mechanism of interactions with the bacterial cell envelope. The spatial orientation of amino acid side-chains upon interacting with the bacterial membrane is often fundamental for the activity of AMPs^56^. The compounds **B1**, **23** and **26** all contain seven residues, which is too short to display α-helical structure^57^. Therefore, they most likely form random coils. Furthermore, **26** contains D-amino acids, which are known to disrupt α-helix structure^58^. Finally, the presence of even a single peptoid residue in an α-helix has been reported to result in a significant reduction of the helical content^56^.

Similarities and differences among bacterial membranes of different species are crucial in determining the spectrum of activity of amphipathic AMPs^59^. Since the structure of **B1**, **23** and **26** are closely related, we speculate that the mechanism of action of **23** and **26** is similar to that of **B1**. Compound **B1** is an L-peptide/peptoid hybrid, **23** is a full L-peptide containing non-canonical amino acids, whilst **26** is an L/D-peptide hybrid (Fig. 1). The affinity of antimicrobial peptides for bacterial cells is due to their amphiphilic properties (hydrophobic and positively charged). The interaction with the bacterial membrane is regulated, for both peptides and peptoids, by their hydrophobicity. Also, the presence of peptoid residues in the backbone of **B1** may correspond to a higher structural flexibility and a more cell-penetrating action of B1. On the other hand, the peptides **23** and **26** may have a more pronounced membrane-disrupting activity, which may correlate with the faster killing effect observed against *S*. *pseudintermedius*.

Veterinary medicine needs antimicrobials which are tuned to a veterinary spectrum and are not shared with human medicine. These considerations are in line with the “One Health” view of infectious diseases, which acknowledges that humans and animals share the same pool of bacterial pathogens. Moreover, MRSP has been isolated in humans, highlighting its zoonotic potential and therapeutic challenge^60^. Therefore, it is in the interest of veterinary medicine and public health that novel antimicrobial agents target *P*. *aeruginosa* and *S*. *pseudintermedius* in companion animals selectively and not closely related species within a genus, including *S*. *aureus*.

In this perspective, we have characterized a novel peptide-peptoid hybrid **B1** with antimicrobial activity against both *S*. *pseudintermedius* and *P*. *aeruginosa*, the main cause of pyoderma in dogs. A structure-activity study identified two compounds, **23** and **26**, with potent activity against the aforementioned species, paired with poor activity against the closely related *S*. *aureus*. Our results represent a first step towards the design of peptide-based antimicrobials with a pathogen-targeted spectrum, even within a genus. Such investigation deserves further exploration towards the rationale design of drugs selective for veterinary pathogens.

## Materials and Methods

### Synthesis of peptides and peptidomimetics

The synthesis of the peptides and peptidomimetics was performed as described by Oddo *et al*.^61^. Briefly, peptides were synthesized by Fmoc solid phase peptide synthesis (SPPS). Peptidomimetics were prepared by a combination of the above and sub-monomer peptoid synthesis. Following TFA-cleavage, precipitation in ether and lyophilisation, the compounds were purified (>95%) by preparative HPLC and the purity was determined through analytical HPLC. The identity of each compound was verified by MALDI-TOF-MS. The compounds used in this study are shown in Supplementary Information (Table S1).

### Antimicrobial susceptibility testing

Activity of B1 was tested on 50*P*. *aeruginosa* isolates and 57*S*. *pseudintermedius* isolates (including 7 MRSP) that had been isolated in the diagnostic laboratory Sund Vet Diagnostik (University of Copenhagen) from various infections in dogs between 2009 and 2011. Representative MRSP (C22963), MSSA, and *P*. *aeruginosa* (26314) were used for testing the first set of B1 analogues.

For antibacterial spectrum the following strains were used: *Corynebacterium auriscanis*, 31551, (54 C6, dog, ear, 2013); *Enterococcus faecalis*, 27404, (17 C7, dog, wound, 2011); *Enterococcus faecium*, 30951, (24 C1, dog, ear, 2013); *Streptococcus canis*, 26740-1, (14 H1, dog, ear, 2010); *Staphylococcus aureus*, 27266, (16 G9, dog, skin, 2010); *Staphylococcus aureus*, 27266, (16 G9, dog, skin, 2010); *Staphylococcus pseudintermedius* 22963, (3 B9, dog, 2007). *Acinetobacter baumannii*, 27065, (16 D1, dog, wound, 2010); *E*. *coli*, 30235, 23 A6, dog, wound, 2012; *Klebsiella pneumoniae*, 26233, (11 H5, dog, wound, 2010); *Pasteurella canis*, 31096, (24 C8, dog, skin, 2013); *Proteus mirabilis*, 25178, (9 A4, dog, ear, 2009); *Pseudomonas aeruginosa* 26314, 12 C5, dog, urine, 2010).

For testing the selectivity of **23** and **26** against clinical isolates of *S*. *pseudintermedius* vs *S*. *aureus the* following strains were used.

#### *S*. *aureus* strains

25054, (8 G6, dog, wound, 2009); 27266, (16 G9, dog, skin, 2010 also used in exp above); 28264,(20 B1, dog, wound, 2011); 30935, (24 B9, dog, joint, 2013); 36968, (61 A9, dog, wound, 2016); 37595,(65 D2, dog, joint, 2016); 37708-2, (66 C6, dog, skin, 2016); 38200, (68 E9, dog, skin, 2016); 38565-1, (70 A5, dog, skin, 2017); 38841, (70 G5, dog, urine, 2017).

#### *S*. *pseudintermedius* strains

26071, (11 E5, dog, skin, 2009); 26092-2, (11 E4, dog, skin, 2009); 26959, (15 F8, dog, wound, 2010); 27364, (17 A7, dog, wound, 2011); 27382, (17 B8, dog, ear, 2011); 27382, (17 B8, dog, ear, 2011); 31524, (54 C3, dog, ear, 2013); 33228, (55 E2, dog, skin, 2014); 35890, (59 B8, dog, ear, 2015); 37526-1, (65 B4, dog, skin, 2016); 37535-1, (65 B6, dog, skin, 2016); 37535-1, (65 B6, dog, skin, 2016); 38637, (70 C3, dog, wound 2017); 38820, (70 F9, dog, urine 2017).

MIC determination was performed by broth microdilution according to the Clinical and Laboratory Standard Institute (CLSI, M31-A3, 2008)^62^. In brief, each bacterial strain was diluted to concentration of 5 × 10^5^ CFU/mL in Mueller Hinton broth II (MHB II) media (Oxoid) and added to a two-fold serial dilution of peptides and peptide-peptoid hybrids concentrations ranging from 1 to 64 µg/ml in 96-well plates (Nunc Internationals, Rochester, NY). The MICs were determined as a lowest concentration showing no visible growth after incubation for 18 hours at 37 °C. Experiments were performed in triplicates on two different days.

### Time kill curves

Time kill assays were performed in triplicate, meaning that every value is the average of three different samplings. Time–kill kinetic assays were performed using *P*. *aeruginosa* (26314), MRSP E104 and/or MRSP C22963 to determine the cell killing activity of **B1** and two of its most promising analogues (compounds **23** and **26**) based on prior antimicrobial susceptibility testing. The method reported by Blondeau *et al*.^63^ was followed with minor modifications. Briefly, the assay was performed in MHB II with concentrations corresponding to 0.5, 1, 2 and 4 times the MIC of the strain. After 0, 15, 30, 60, 120, 180 and 300 min of growth, 100 μL aliquots were collected and 10-fold serially diluted. Twenty μL of cell suspension from each dilution were spotted in triplicate on blood agar plates followed by 16–18 h incubation at 37 °C and determination of colony forming units (CFU).

### Haemolytic activity

The EC_10_ (10% maximal effective concentration), EC_50_ (half maximal effective concentration) values and the percentage of haemolysis at 150 μM were determined for all compounds as previously described^64^. Briefly, two-fold serial dilutions (2.35 to 150 μM) of compounds in phosphate buffer saline (PBS) were mixed with equal volume of 0.5% v/v suspension of fresh human red blood cells (RBC) in the same buffer. After 1 h incubation at 37 °C, plates were centrifuged and aliquots of the supernatants were transferred to clear 96-well plates. Absorbance at 414 nm was measured and normalized using a negative (PBS, 0%) and a positive (melittin, 100%) control. The EC_10_ and EC_50_ are concentrations at which 10% and 50% of RBC were lysed, respectively, as interpolated graphically by the y-axis intersection of the plotted data.

### Macromolecule biosynthesis rate

Macromolecule biosynthesis rate was measured in MRSP E104 following a protocol adapted from Ling *et al*.^65^. Briefly, E104 overnight culture was sub-cultured 1:100 in MHB II and grown up to OD 0.2 at 600 nm. Cells were pelleted down by centrifugation and resuspended in fresh medium followed by incubation for 20 min with **B1** or nisin at 1.5 µg/ml and 1.25 µg/ml, respectively, and radiolabeled precursor: (50µCi) 3H-Thymidine (PerkinElmer) and (5µCi) 3H-glucosamine hydrochloride per ml for DNA and cell wall, respectively. A positive control without antimicrobial was maintained. After incubation, samples were precipitated with equal volume of cold 30% TCA (Sigma) on ice. Precipitates were filtered on a membrane filter and subsequently subjected to two washes of cold 15% TCA and two washes of cold water using vacuum manifold. Subsequently, filters were air dried overnight and then transferred to 10 ml scintillation vials. Finally, scintillation fluid (3 ml) was added to each vial and ^3^H count was taken in Beckman Coulter LS6500 liquid scintillation counter for one minute. The radioactive counts of the control samples were considered to have 100% precursor incorporation and macromolecule synthesis. The percentage rates of the antimicrobial-exposed samples were calculated accordingly. Each experiment was performed with replicates and the average rates of incorporation were plotted.

### DiSC3(5) fluorescence-based membrane potential study

Freshly sub-cultured E104 cells were labelled with 1 µM 3,3-Dipropylthiadicarbocyanine iodide [DiSC3(5)] (Sigma) in MHB II. The fluorescence spectra of labelled cells were plotted in the LS50B luminescence spectrometer (PerkinElmer) at excitation/emission wavelengths 546 nm/573 nm using time drive application of FLWINLAB software. After reading an initial stable emission spectra of DiSC3(5), labelled cells were treated with B1 or nisin or protonophore CCCP at 1, 1.5 and 1.25 µg/ml, respectively and the change of fluorescence over time was recorded. The increment of DiSC3(5) fluorescence upon addition of antimicrobials or CCCP (FUafter-treatment – FUbefore-treatment) was plotted. This experiment was performed twice and each time with two technical replicates.

### Flow cytometry analysis of antimicrobial-exposed cells

Freshly grown cultures (OD 0.2 at 600 nm) of MRSP E104 were exposed to 3 µg/ml of **B1** or 2.5 µg/ml of nisin for 1 h and diluted 1:10 in flow cytometry analysis buffer (PBS supplemented with 1 mM EDTA, 0.01% Tween 20 and filtered by 0.22 µm membrane). Diluted cells were stained with 420 nM thiazole orange (TO, Sigma) and 48 µM propidium iodide (PI, Sigma) and analysed using BD ACCURI C6 flow cytometer (BD Biosciences). Unstained cells were used as negative control. Finally, the differentially labelled bacterial populations in antimicrobial-exposed cultures were plotted under FL3(red)/FL1(green) axis using the instrument’s software and categorised into four different conditions: P1 (unstained), P2 (dead cells), P3 (injured cells) and P4 (live cells).

## Supplementary information

Greco et al Supplementary InformationR2
