# Publisher Correction: Characterization, mechanism of action and optimization of activity of a novel peptide-peptoid hybrid against bacterial pathogens involved in canine skin infections

**DOI:** 10.1038/s41598-019-44436-4

**Published:** 2019-06-04

**Authors:** Ines Greco, Agnete Plahn Emborg, Bimal Jana, Natalia Molchanova, Alberto Oddo, Peter Damborg, Luca Guardabassi, Paul R. Hansen

**Affiliations:** 1 Department of Drug Design and Pharmacology, Faculty of Health and Medical Sciences, University of Copenhagen, Universitetsparken 2, 2100 Copenhagen, Denmark; 2 Present Address: Department of Food Science, Faculty of Science, University of Copenhagen, Rolighedsvej 26, 1958 Frederiksberg, Denmark; 3 Present Address: Novo Nordisk, Brennum Park 1, 3400 Hilleroed, Denmark; 4 Department of Veterinary and Animal Sciences, Faculty of Health and Medical Sciences, University of Copenhagen, Stigbøjlen 4, 1870 Frederiksberg C, Denmark; 5 Present Address: Roskilde University, Department of Science and Environment, 4000 Roskilde, Denmark; 6 Present Address: Novo Nordisk A/S, Krogshøjvej 44, 2820 Bagsværd, Denmark; 7 Department of Pathobiology and Population Sciences, The Royal Veterinary College, Hawkshead Lane, North Mymms, AL9 7TA Hatfield, Herts United Kingdom

Correction to: *Scientific Reports* 10.1038/s41598-019-39042-3, published online 06 March 2019

In the original version of this Article, Paul R. Hansen was incorrectly affiliated with ‘Department of Veterinary and Animal Sciences, Faculty of Health and Medical Sciences, University of Copenhagen, Stigbøjlen 4, 1870, Frederiksberg C, Denmark’. The correct affiliation is listed below.

Department of Drug Design and Pharmacology, Faculty of Health and Medical Sciences, University of Copenhagen, Universitetsparken 2, 2100, Copenhagen, Denmark

This error has now been corrected in the PDF and HTML versions of the Article.

