# Supplementary material for: Characterization, mechanism of action and optimization of activity of a novel peptide-peptoid hybrid against bacterial pathogens involved in canine skin infections
Source: Sci Rep. 2019 Mar 6;9:3679. doi: 10.1038/s41598-019-39042-3 (PMC6403271; doi:10.1038/s41598-019-39042-3)
Supplement: Supplementary file 1 — Greco et al Supplementary InformationR2 [file 41598_2019_39042_MOESM1_ESM.docx]

**Supplementary Information**

**Characterization, mechanism of action and optimization of activity of a novel peptide-peptoid hybrid against bacterial pathogens involved in canine skin infections.**

Ines Greco^1,2^, Agnete Plahn Emborg^1,3^, Bimal Jana^4^, Natalia Molchanova^1,5^, Alberto Oddo^1,6^, Peter Damborg^4^, Luca Guardabassi^4^ and Paul R. Hansen*^1^

^1^Department of Drug Design and Pharmacology, Faculty of Health and Medical Sciences, University of Copenhagen, Universitetsparken 2, 2100, Copenhagen, Denmark

^2^Present address: Department of Food Science, Faculty of Science, University of Copenhagen, Rolighedsvej 30, 1958, Frederiksberg, Denmark

^3^Present address: Novo Nordisk, Brennum Park 1, 3400 Hilleroed, Denmark
^4^Department of Veterinary Disease Biology, Faculty of Health and Medical Sciences, University of Copenhagen, Stigbøjlen 4, 1870, Frederiksberg C, Denmark

^5^Present address: Roskilde University, Department of Science and Environment, 4000 Roskilde, Denmark

^6^Present address: Novo Nordisk A/S, Novo Nordisk Park 1, 2760 Maaloev, Denmark.

Corresponding Author

Paul R. Hansen

Telephone: +4535336625

Fax: +4535336041

e-mail: prh@sund.ku.dk

**Content:**

**S1: Structure, molecular mass, HPLC purity and Retention Time of compounds used in this study.**

**S2: Synthesis of peptides and peptidomimetics**

**S3.1: Reagents**

**S3.2: Peptide and Peptidomimetic Synthesis**

**S3.3: Product Cleavage**

**S3: Reverse-Phase HPLC and MALDI-TOF-MS**

**S4.1: Characterization by analytical HPLC**

**S4.2: Purification by Preparative HPLC**

**S4.3: Characterization by Matrix-Assisted Linear-Desorption-Ionization Time-Of-Flight mass spectrometry.**

**S4. Analytical HPLC chromatograms**

**S5: MIC distribution of MRSP, MSSP and *P. aeruginosa***

**S6: High-throughput growth curves**

**S7: Selectivity of 23 and 26 against *S. aureus* vs *S. pseudintermedius* strains**

**Supplementary Table S1: Structure, molecular mass, HPLC purity and retention time (RT) of compounds used in this study.**

| **ID** | **STRUCTURE** | **Mass Calc´d** | **Mass Obs´d** | **% HPLC purity** | **HPLC RT/min** |
| --- | --- | --- | --- | --- | --- |
| **B1** |  | 1070.394 | 1071.566 | 99.7 | 17.8 |
| **2** |  | 1056.367 | 1057.565 | 99.0 | 17.7 |
| **3** |  | 1056.367 | 1057.537 | 99.5 | 17.8 |
| **4** |  | 1056.367 | 1057.431 | 98.6 | 17.7 |
| **5** |  | 1056.367 | 1057.432 | 95.3 | 17.7 |
| **6** |  | 1056.367 | 1057.569 | 99.6 | 17.0 |
| **7** |  | 1056.367 | 1057.453 | 95.7 | 17.2 |
| **8** |  | 1056.367 | 1057.269 | 98.1 | 17.0 |
| **9** |  | 1056.367 | 1057.565 | 99.0 | 17.3 |
| **10** |  | 1056.367 | 1057.655 | 99.3 | 16.2 |
| **11** |  | 1056.367 | 1057.407 | 99.6 | 16.2 |
| **12** |  | 1056.367 | 1057.526 | 99.7 | 16.1 |
| **13** |  | 1056.367 | 1057.196 | 97.7 | 16.1 |
| **14** |  | 1056.367 | 1057.335 | 97.8 | 16.3 |
| **15** |  | 1056.367 | 1057.369 | 98.8 | 17.6 |
| **16** |  | 1056.367 | 1057.032 | 97.6 | 17.8 |
| **17** |  | 1056.367 | 1057.592 | 99.3 | 17.0 |
| **18** |  | 1056.367 | 1057.508 | 98.5 | 15.8 |
| **19** |  | 1056.367 | 1057.555 | 99.2 | 16.2 |
| **20** |  | 1056.367 | 1057.426 | 98.0 | 15.7 |
| **21** |  | 1056.367 | 1057.401 | 99.8 | 16.1 |
| **22** |  | 1172.366 | 1057.543 | 95.2 | 16.6 |
| **23** |  | 1056.367 | 1057.374 | 97.7 | 17.4 |
| **24** |  | 1056.367 | 1057.615 | 95.1 | 16.3 |
| **25** |  | 1072.366 | 1073.562 | 98.7 | 16.6 |
| **26** |  | 1056.367 | 1057.523 | 98.3 | 17.3 |
| **27** |  | 1072.366 | 1073.352 | 97.7 | 16.8 |
| **28** |  | 1072.366 | 1073.277 | 95.5 | 16.6 |
| **29** |  | 1056.367 | 1057.331 | 98.8 | 17.6 |

**S2: Synthesis of peptides and peptidomimetics.**

**S2.1**. **Reagents.** HypoGel200RAM (loading: 0.62 mmol/g), TentaGelSRAM (0.2 mmol/g), TFA (trifluoroacetic acid), piperidine and Fmoc (9-fluorenylmethyloxycarbonyl)-protected amino acids were purchased from Iris-Biotech GmbH. The compounds 1-naphthylmethylamine, 4-methylbenzylamine, benzylamine, butylamine, N,N-diisopropylcarbodiimide (DIPCDI), N,N-diisopropylethylamine (DIEA), 3,3′- triisopropylsilane (TIS) were from Sigma. 1-hydroxy-7-azabenzotriazole (HOAt) and 1-[Bis(dimethylamino)methylene]-1H-1,2,3-triazolo[4,5-b]pyridinium 3-oxid hexafluoro-phosphate (HATU) were from GL Biochem Shanghai. Acetic acid, DMF (dimethylformamide), DCM (dichloromethane), ACN (acetonitrile) were from WWR. All reagents and solvents were used without further purification. Disposable 5-ml polypropylene reactors fitted with a PTFE filter were acquired from Thermo Scientific. Cellulose membrane filter (cat no. AAWG0250C) was purchased from Millipore.

**S2.2.** **Synthesis of peptides and peptide-peptoid hybrids**. Peptide Synthesis: Peptides were synthesized manually on 100 mg of TentaGelSRAM (0.2 mmol/g) or HypoGel200RAM (loading 0.62mmol/g) according to standard 9-fluorenylmethyloxycarbonyl (Fmoc) solid-phase peptide synthesis procedures. The syringes were fitted with fritted PTFE filters and disposable pipette tips, which allowed them to stand vertically in an in­house made suction plate, connected to a 2 L Büchner flask and a vacuum pump. Fmoc-protected amino acids (5 equiv.) were coupled to the growing resin using HATU/HOAt)/diisopropyleththylamine (1:1:2) for 1.5 hours in DMF. The coupling of the first amino acid and every amino acid after a large aromatic amino acid was followed by a recoupling. The Fmoc protecting group was removed in three repetitions of adding 20 % piperidine in DMF (3 mL, 4 min) to each syringe. The resin was then washed twice with DMF in between. The resin was finally washed 10 times with DMF (~3 mL) after the last deprotection.

Peptoid synthesis: Peptoid residues were coupled to the resin by adding 0.6 M bromoacetic acid (10 eq) in DMF and 3.2 M DIPCDI (10 eq). The support was agitated for 30 min and drained. After washing with DMF, the side chain was introduced by nucleophilic substitution of the halide with a primary amine (40 eq) in DMF and agitated for 2 h.

**S2.3**.**Product cleavage.** Following synthesis, the resin was washed 10 times with DMF, then 5 times with EtOH and left to dry in vacuo and freeze-dried overnight. The syringes were fitted with pressure caps. A solution of TFA:H_2_O:TIS (95:2.5:2.5) (~3 mL) was added to the resin and the opening of the syringes were covered with parafilm. The syringes were left vertically for 2 h. The cap on the syringe was removed and the cleavage solution containing the peptide was collected in the prepared cryotube. The resin was further washed twice with TFA:H_2_O:TIS (95:2.5:2.5) (2 mL) which was also collected in the cryotube. Finally TFA was evaporated by N2 leaving a volume of approximately 300 μL of peptide-solution. Cold diethyl ether (4 mL) was added to the remaining peptide solution in the cryotubes. The lid was put back on and the mixture was shaken thoroughly to mix the two phases. The cryotubes were centrifuged at 2,000 rpm for 6 min followed by removal of the supernatant. This was repeated twice, though the last centrifugation was at 4000 rpm. The cryotubes were left open overnight to let residual diethyl ether evaporate. The peptide was dissolved in H_2_O:ACN:TFA (90:10:0.1) (1.5 mL) and freeze-dried overnight.

Crude peptide purity was determined by analytical HPLC using a Waters Symmetry C18 column (4 m particle size, 90 Å pore size) on a Waters system with Empower Pro software. The column was equilibrated with eluent A (0.1% [vol/vol] trifluoroacetic acid in water), and peptide was eluted with a gradient of eluent B (90% [vol/vol] acetonitrile, 10% [vol/vol] water, 0.1% [vol/vol] trifluoroacetic acid). If purity by peak area was less than 95%, the peptide was subjected to purification by preparative HPLC using a Waters XBridge BEH130 C18 column (10 x 250 mm, 5µm) on a Waters system with Empower Pro software. The column was equilibrated with eluent A, and peptide was eluted with a gradient of eluent B. The correct peptide peak was identified using the HPLC UV spectrum and verified by matrix-assisted laser desorption ionization (MALDI)–time of flight (TOF) mass spectrometry (MS) on a Bruker Microflex system using α-cyano-4-hydroxycinnamic acid as matrix .

**S3: Reverse-Phase HPLC and MALDI-TOF-MS.**

**S3.1 Characterization by analytical HPLC.**

Purity was assessed by analytical RP-HPLC column (Waters XBridge™ BEH C18 Column, 130Å, 3.5 µm, 4.6 mm X 150 mm) The system consisted of Waters In-Line Degasser, 600 Controller, 2996 Photodiode Array Detector, 600 Pump and 717plus Autosampler.

Compounds were dissolved in H_2_O:ACN:TFA (90:10:0.1) (1.5 mL). This solution (30 μL) and ACN (80 μL) were transferred into a HPLC vial. A volume of 10-15 μL was injected into the column and analysed in a 30 min run with 9 min delay until next injection. The analysis was performed by gradient elution Method 1 (Table S2) using mobile phases: A: 0.01 % TFA in Milli-Q water and B: 90 % ACN in Milli-Q water + 0.01 %. During the delay 100 % A is used with a flow of 1.5 mL/min. Peaks were detected at 220 nm. The ApexTrack™ algorithm was used for peak integration.

**Supplementary Table S2. Gradient elution Method 1 used for analytical RP-HPLC (30-40 min is as delayed injection and do not show on the chromatogram). % A and % B indicates the percentage of the mobile phases mixed at the given time.**

**Time (min) Flow (mL/min)**

**% A % B**

1.5 100 0

**20** 1.5 30 70

**22** 1.5 0 100

**27** 1.5 0 100

1. 1.5 100 0

#### S3.2 Purification by preparative HPLC

Crude products were dissolved in ACN (150 μL) and Milli-Q water (150 μL) and injected into a preparative RP-HPLC column (Waters XBridge™ Prep BEH C18 Column, 5 µm, 10 X 250 mm) equipped with a guard column (Waters Cartridge Holder PKG 10 x 10 mm). The system consisted of Waters In-Line Degasser, 600 Controller, 996 Photo Array Detector and 600 Pump. The purification was performed by gradient elution *Method 2* (Table S3) using the same mobile phases as for analytical RP-HPLC.

**Supplementary Table S3. Gradient elution *Method 2* used for purification of compounds on preparative RP-HPLC.**

|  | **Time (min)** | **Flow (mL/min)** | **% A** | **% B** |
| --- | --- | --- | --- | --- |
|  |  | 4 | 100 | 0 |
| **1** |  | 6 | 100 | 0 |
| **3** |  | 8.5 | 100 | 0 |
| **5** |  | 8.5 | 90 | 10 |
| **20** |  | 8.5 | 45 | 55 |
| **26** |  | 9 | 0 | 100 |
| **27** |  | 9 | 0 | 100 |
| **30** |  | 9 | 100 | 0 |
| **39** |  | 9 | 100 | 0 |
| **40** |  | 1.5 | 100 | 0 |

For compounds where peaks from impurities were close to or overlapped with the compound peak, Method 3 (Table S4) with a slower gradient was used to better separate the peaks.

**Supplementary Table S4. Gradient elution *Method 3* used for purification of compounds on preparative RP-HPLC.**

|  | **Time (min)** | **Flow (mL/min)** | **% A** | **% B** |
| --- | --- | --- | --- | --- |
|  | **0** | 4 | 100 | 0 |
|  | **1** | 6 | 100 | 0 |
|  | **3** | 9 | 100 | 0 |
|  | **5** | 9 | 90 | 10 |
|  | **20** | 9 | 60 | 40 |
|  | **26** | 9 | 0 | 100 |
|  | **27** | 9 | 0 | 100 |
|  | **30** | 9 | 100 | 0 |
|  | **39** | 9 | 100 | 0 |
|  | **40** | 1.5 | 100 | 0 |

All compounds were purified by a 40 min run. Peaks were detected at 220 nm and 280 nm to separate impurities from the aromatic compounds.The collected fractions were evaporated *in vacuo* and the dry product was transferred to a clean cryotube and freeze-dried overnight.

**S3.3 Characterization by Matrix-assisted Laser Desorption-Ionization Time-of-Flight mass spectrometry**

Verification of molecular mass was achieved by matrix-assisted laser desorption-ionization Time-of-Flight mass spectrometry (MALDI-TOF MS). A droplet (0.5 μL) of a preparative HPLC fraction of the pure compound was applied on the target plate, and allowed to dry completely. Alternatively, a small amount of the dry compound was dissolved in Milli-Q water (700 μL) and ACN (300 μL) reaching a concentration in the range of 0.01-0.1 mg/mL. When dry, a matrix solution (0.5 μL) was added on top of the sample spot and allowed to dry completely. The matrix was prepared by dissolving ACCA (10 mg) in ACN:H_2_O:TFA (500:475:25) (1 mL).The molecular mass was analysed by a Bruker Microflex MALDI-TOF MS using the software flexControl. Data was processed by the software flex Analysis.

**S4.Analytical HPLC chromatograms**

**Analytical HPLC chromatogram of compound B1**


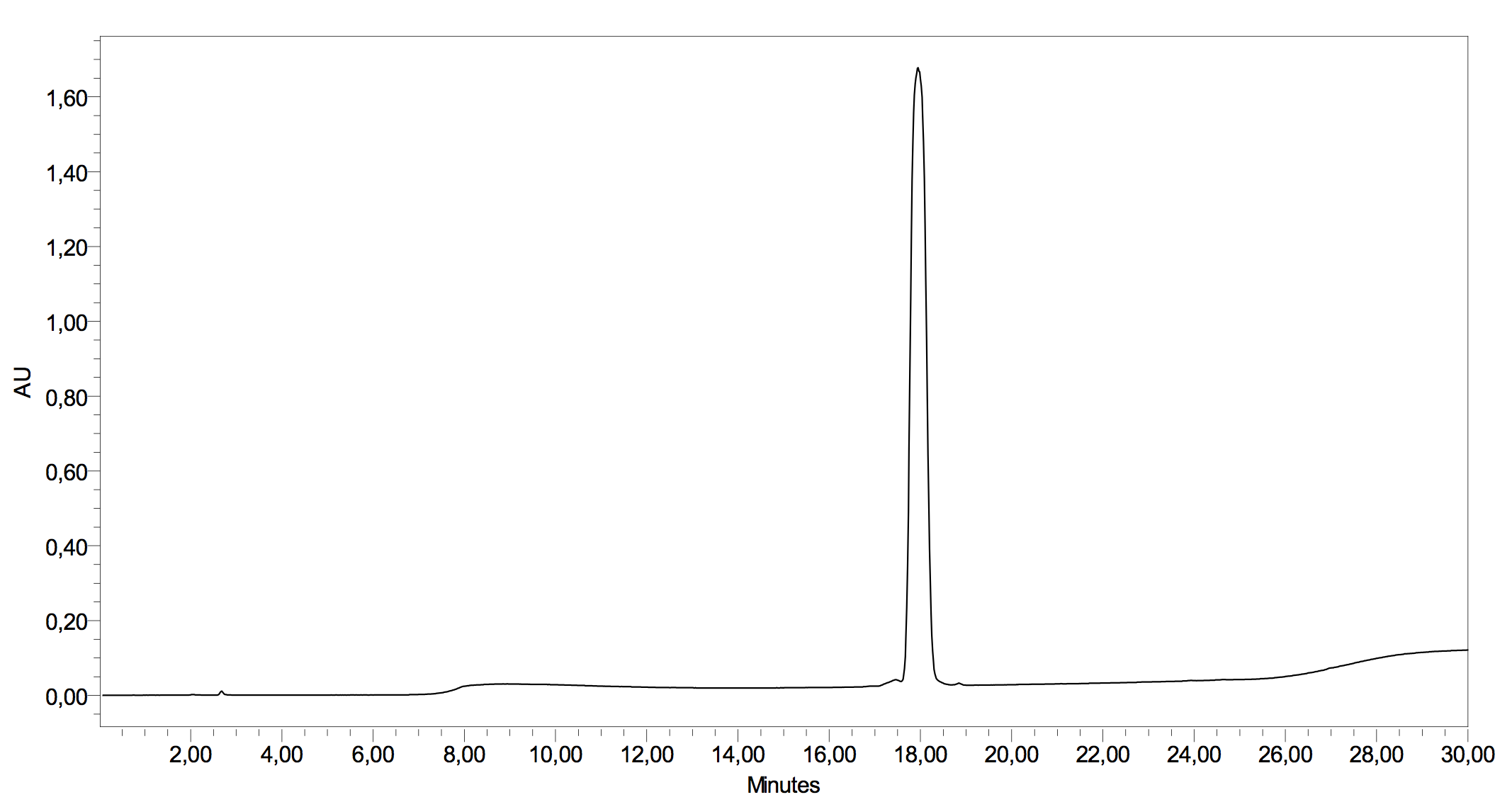


**Analytical HPLC chromatogram of 2**


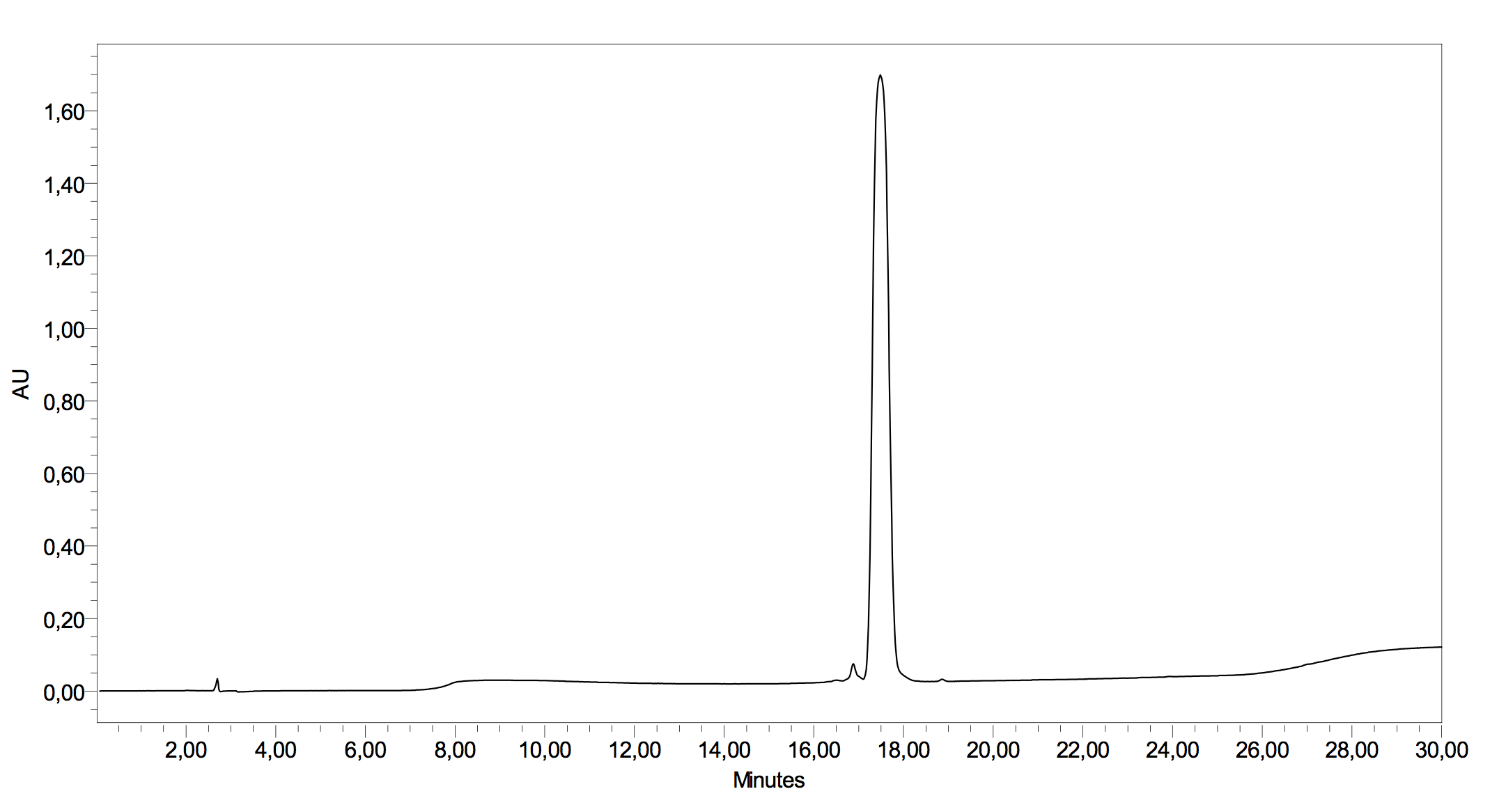


**Analytical HPLC chromatogram of 3**


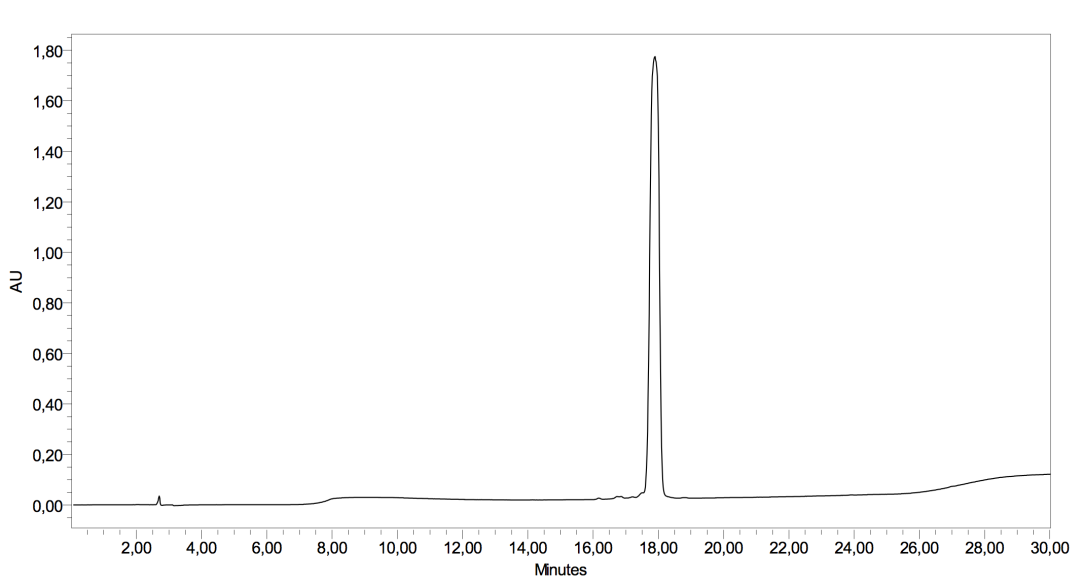


**Analytical HPLC chromatogram of 4**


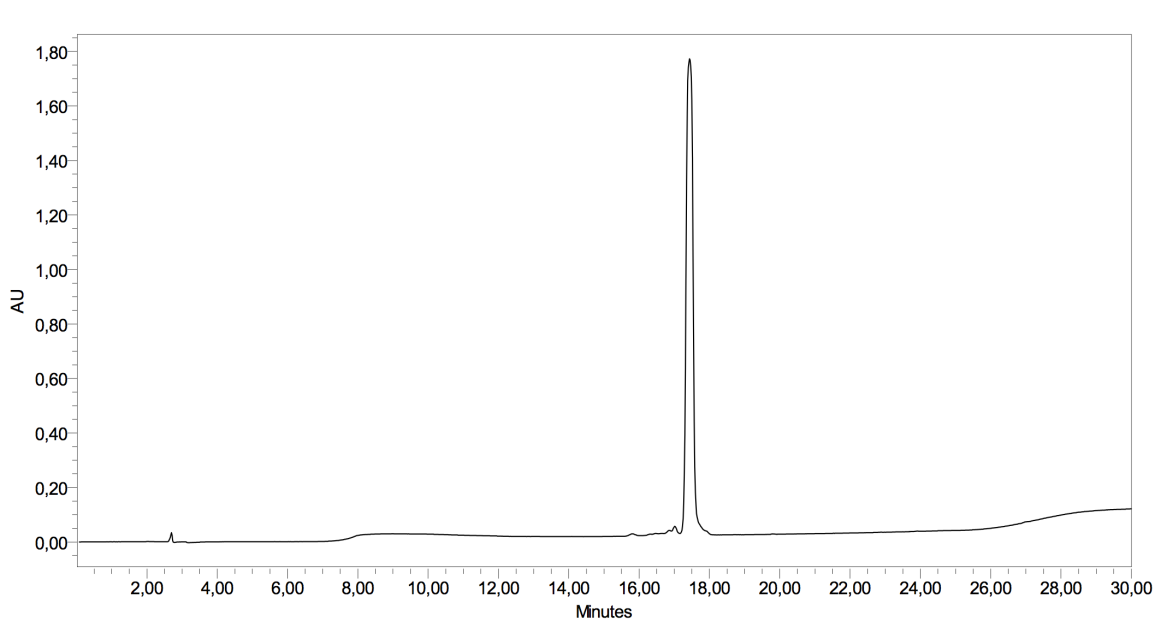


**Analytical HPLC chromatogram of 5**


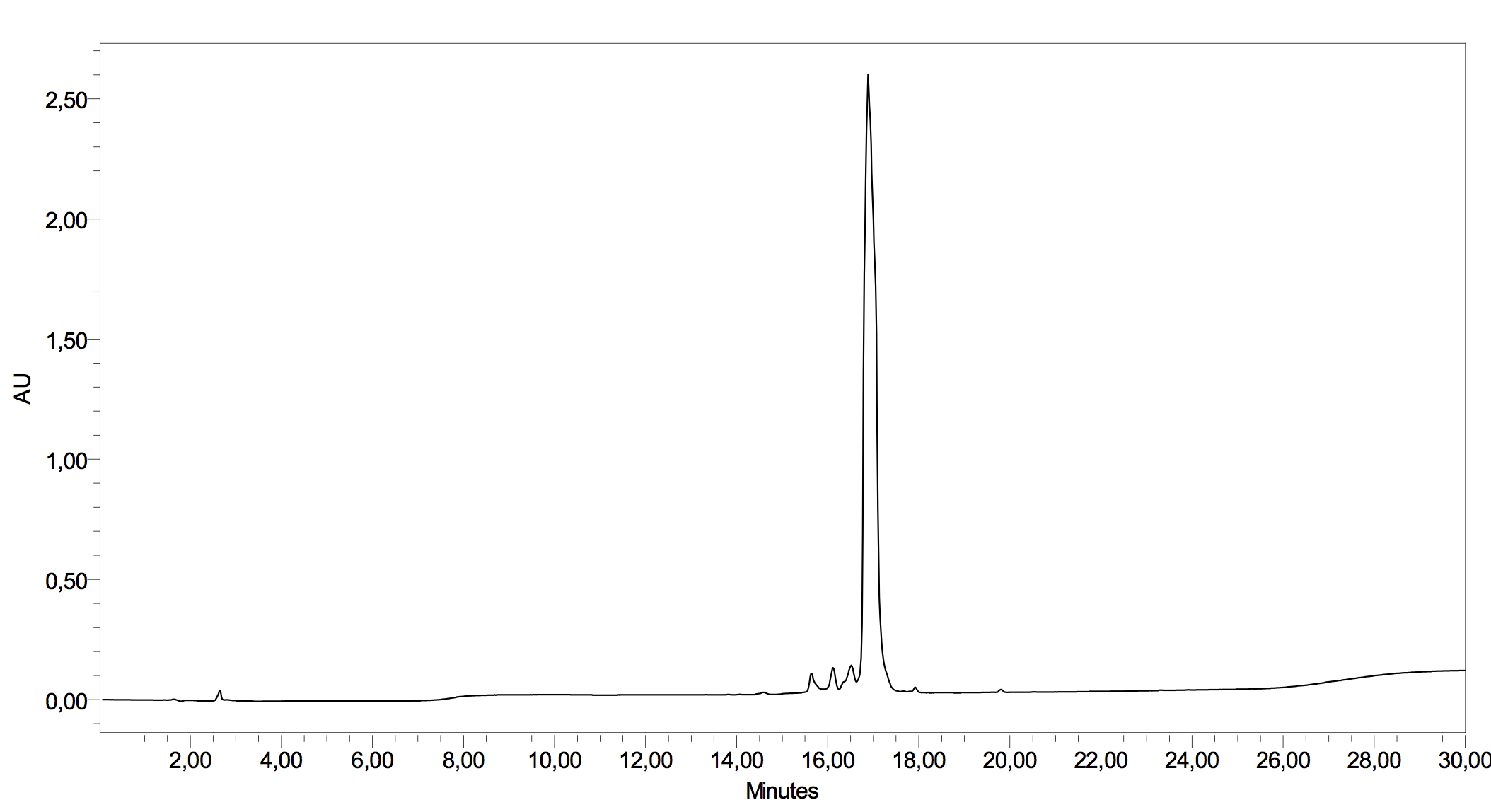


**Analytical HPLC chromatogram of 6**


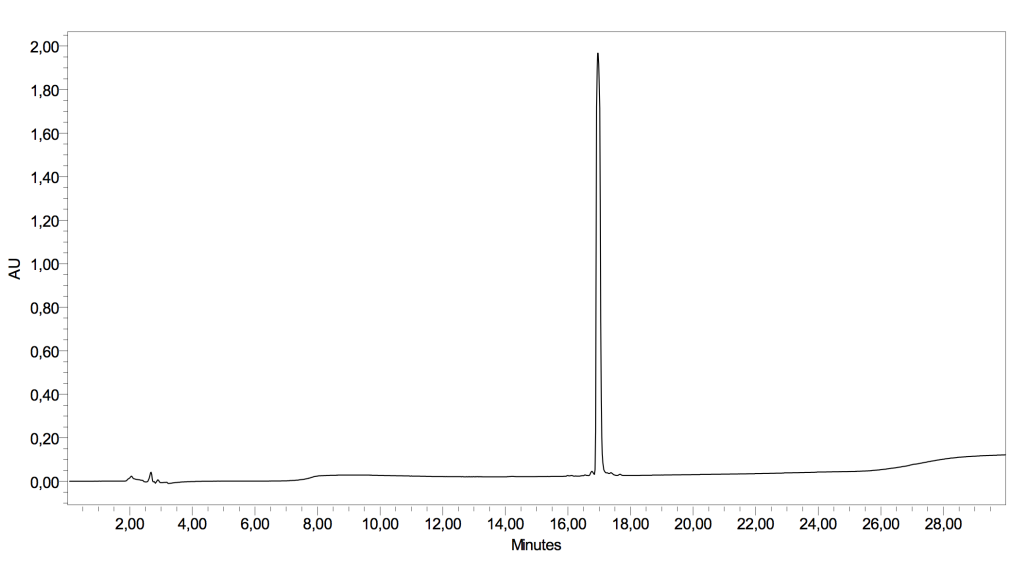


**Analytical HPLC chromatogram of 7**


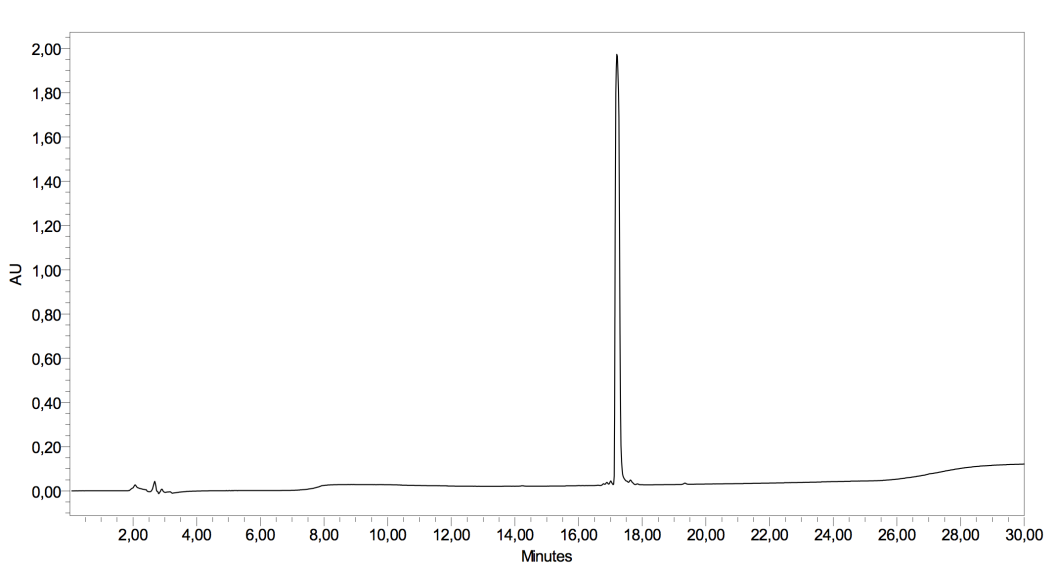


**Analytical HPLC chromatogram of 8**


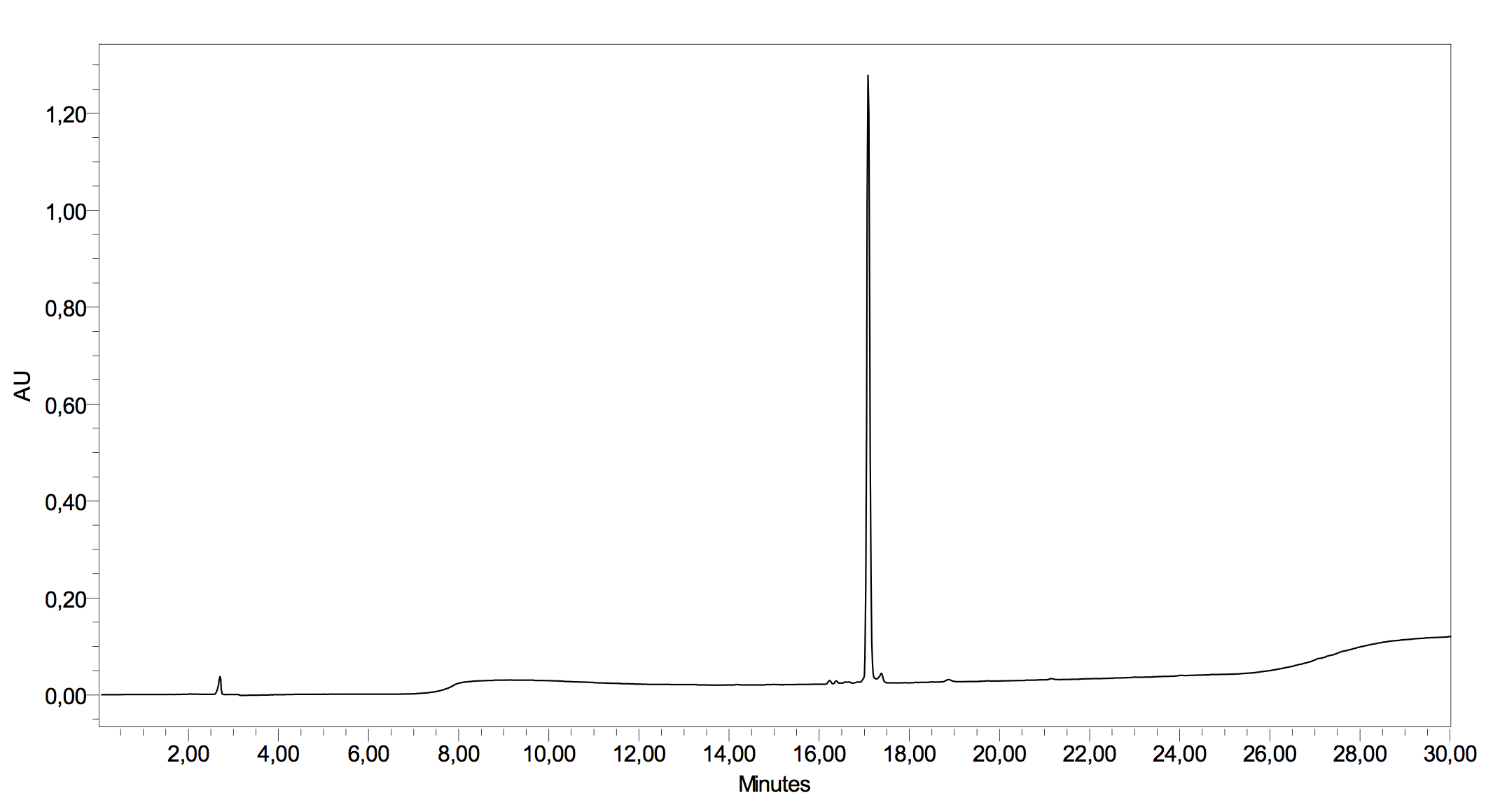


**Analytical HPLC chromatogram of 9**


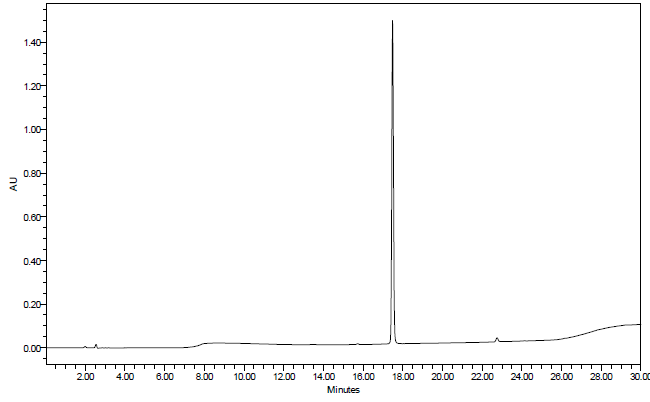


**Analytical HPLC chromatogram of 10**


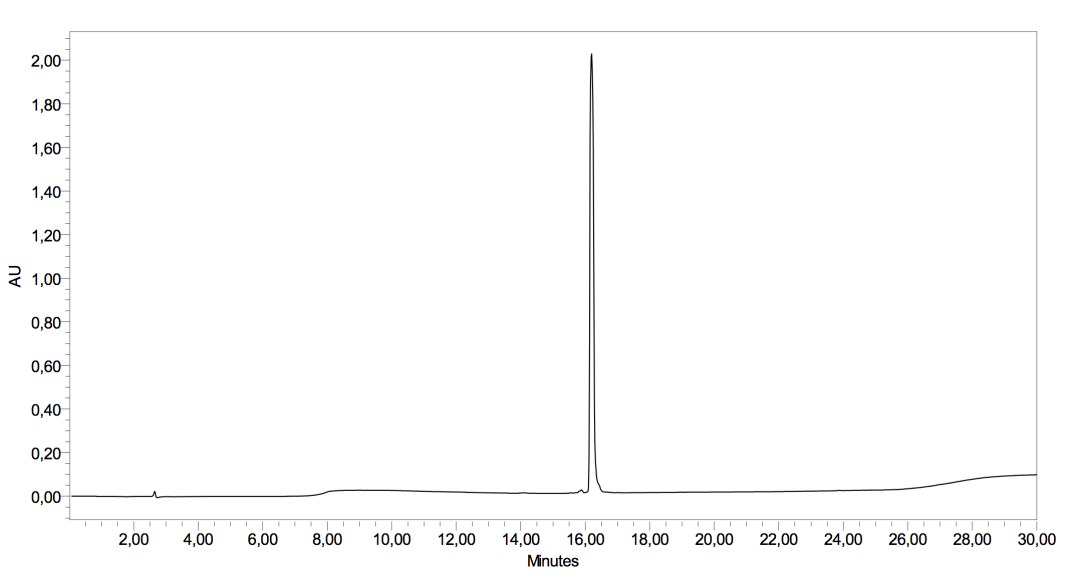


**Analytical HPLC chromatogram of 11**


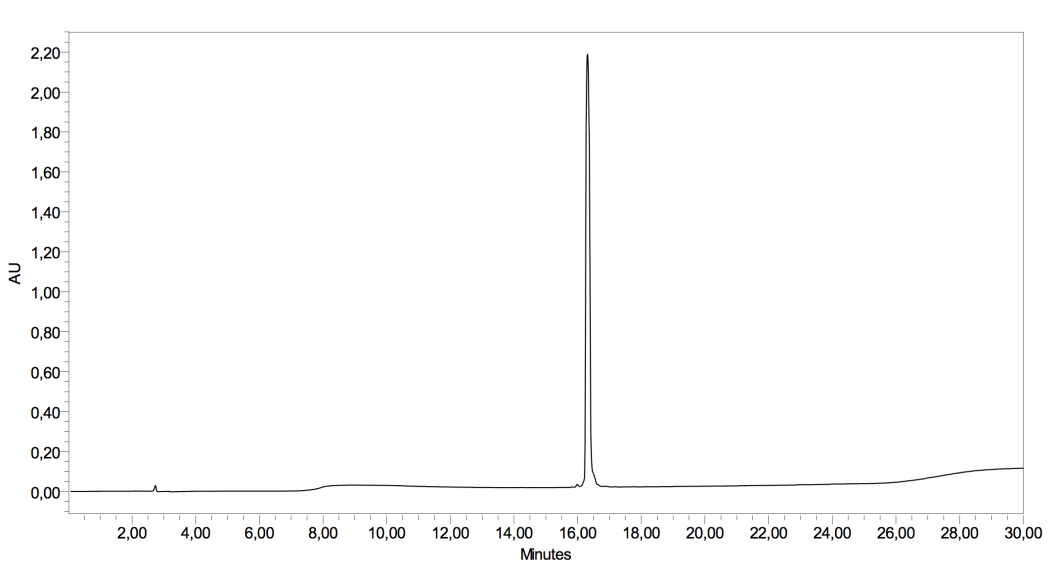


**Analytical HPLC chromatogram of 12**


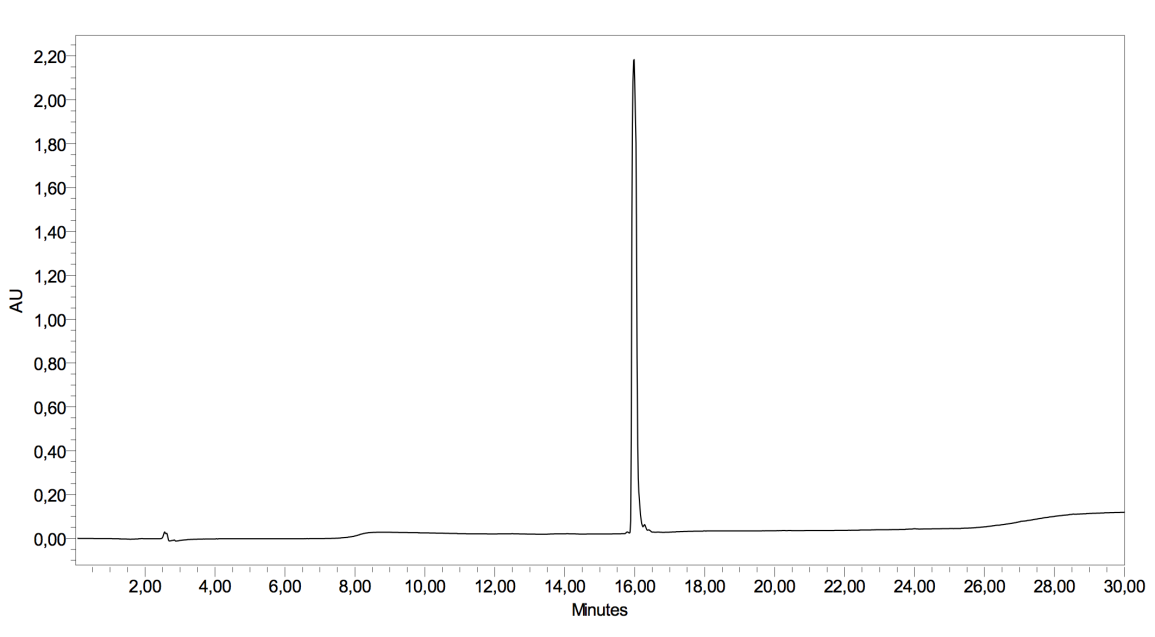


**Analytical HPLC chromatogram of 13**


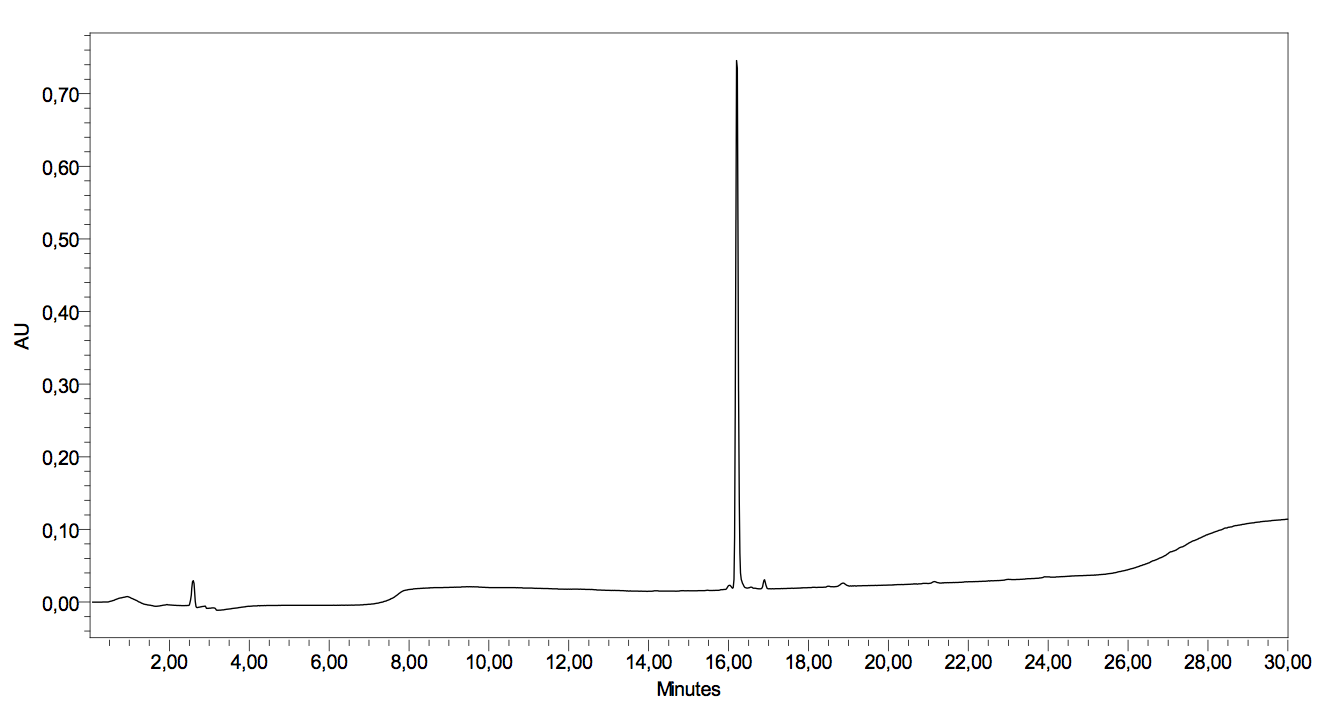


**Analytical HPLC chromatogram of 14**


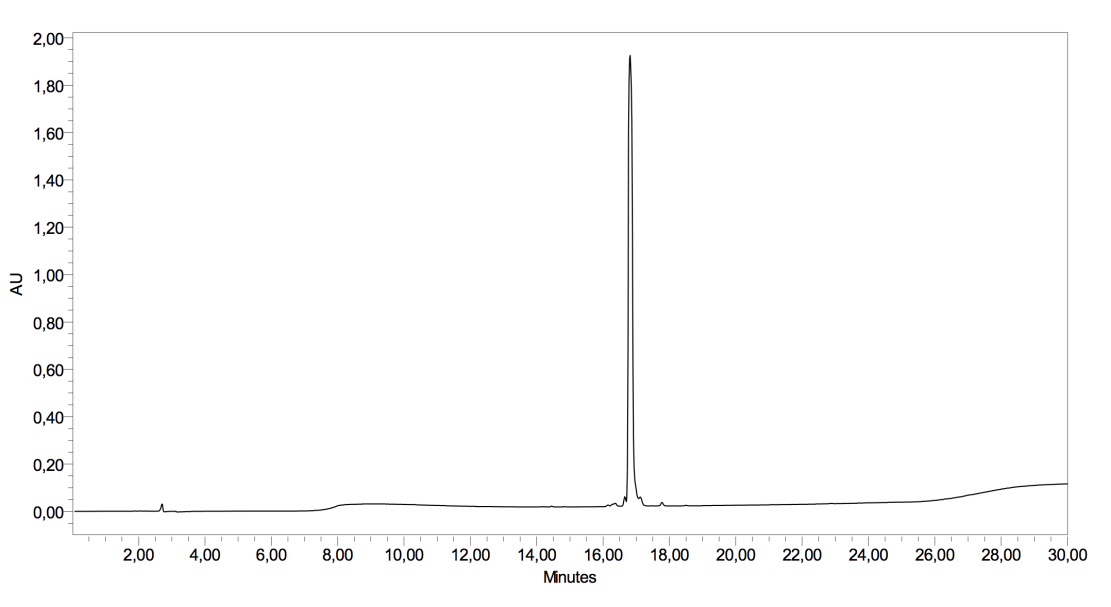


**Analytical HPLC chromatogram of 15**


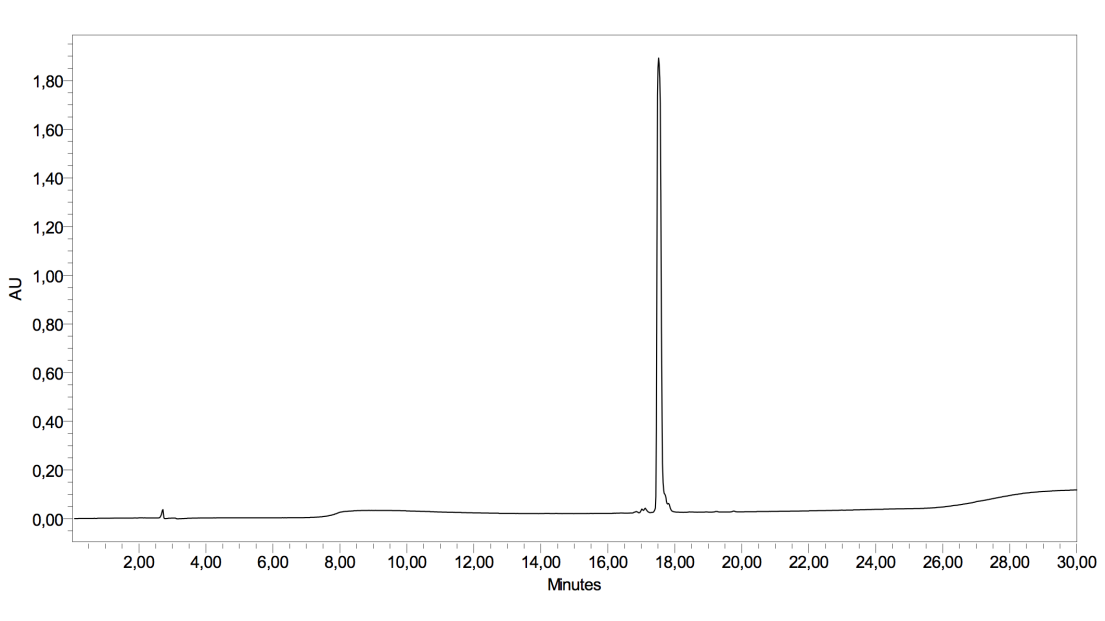


**Analytical HPLC chromatogram of 16**


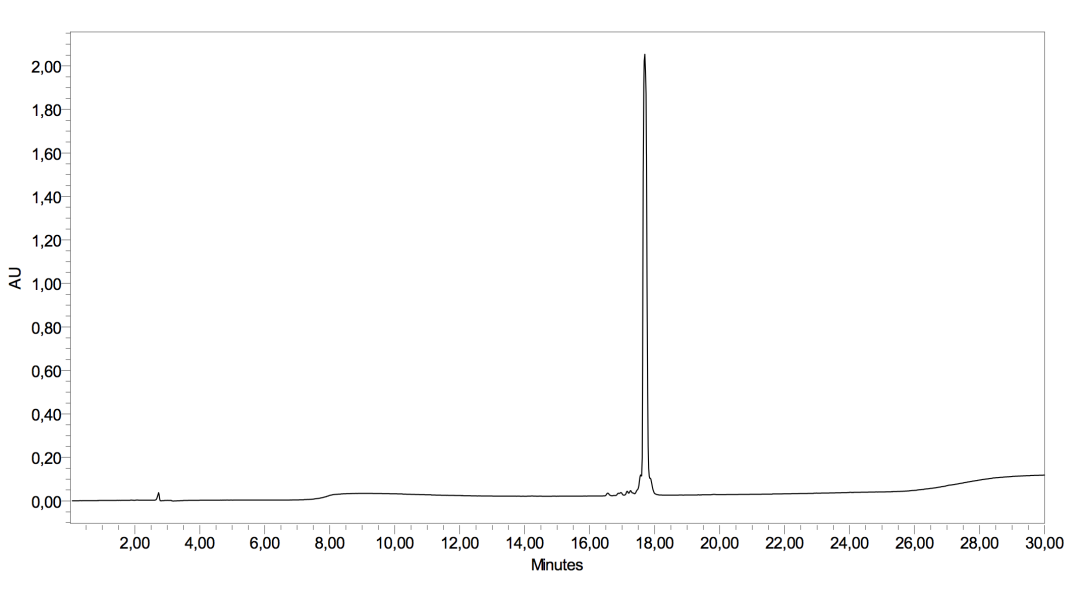


### **Analytical HPLC chromatogram of 17**


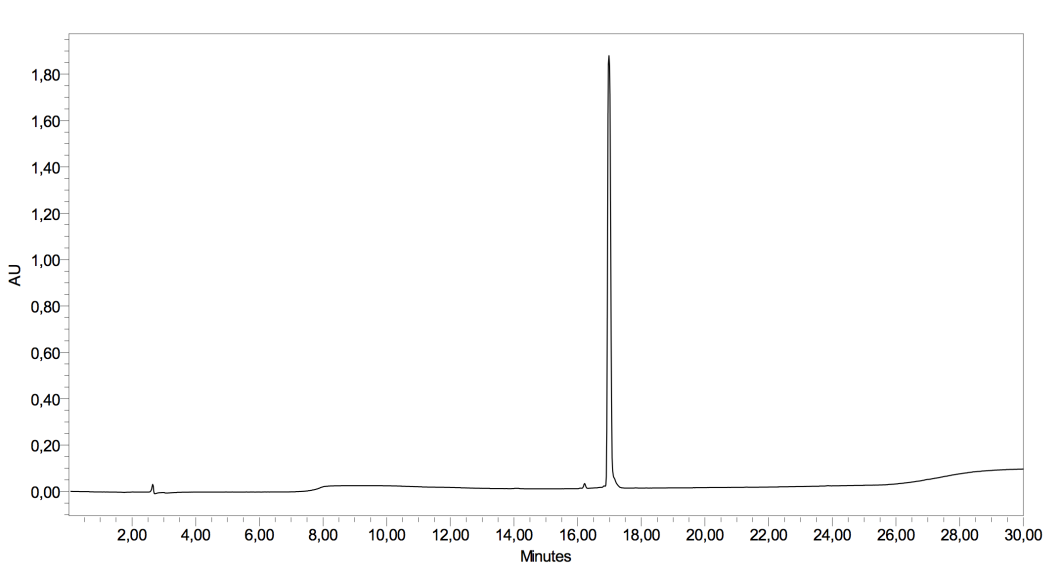


**Analytical HPLC chromatogram of 18**


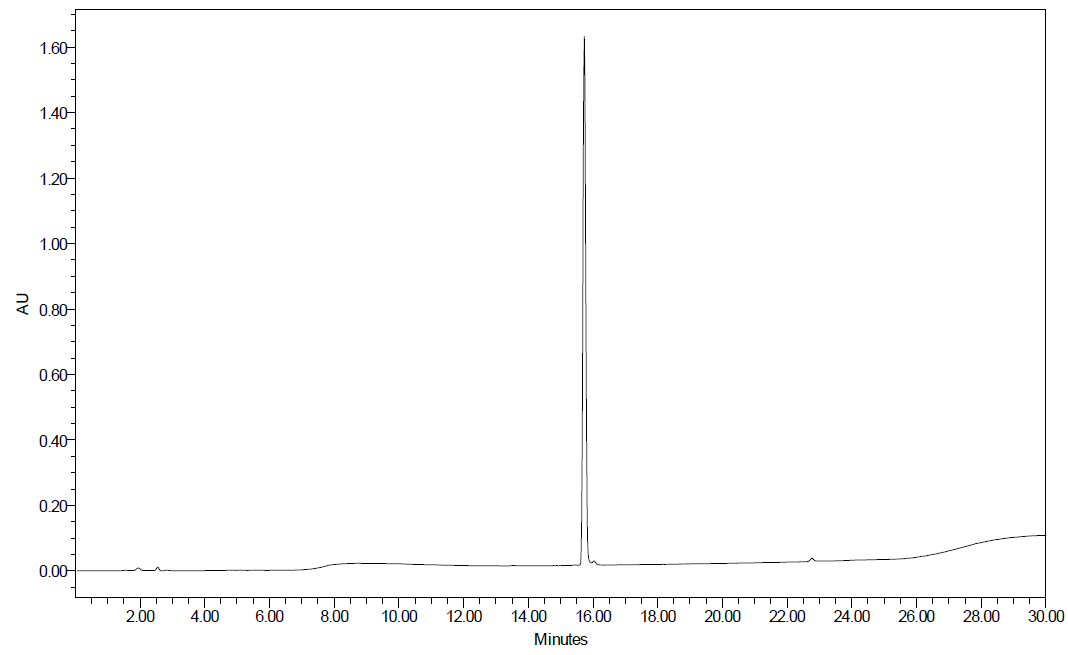


**Analytical HPLC chromatogram of 19**


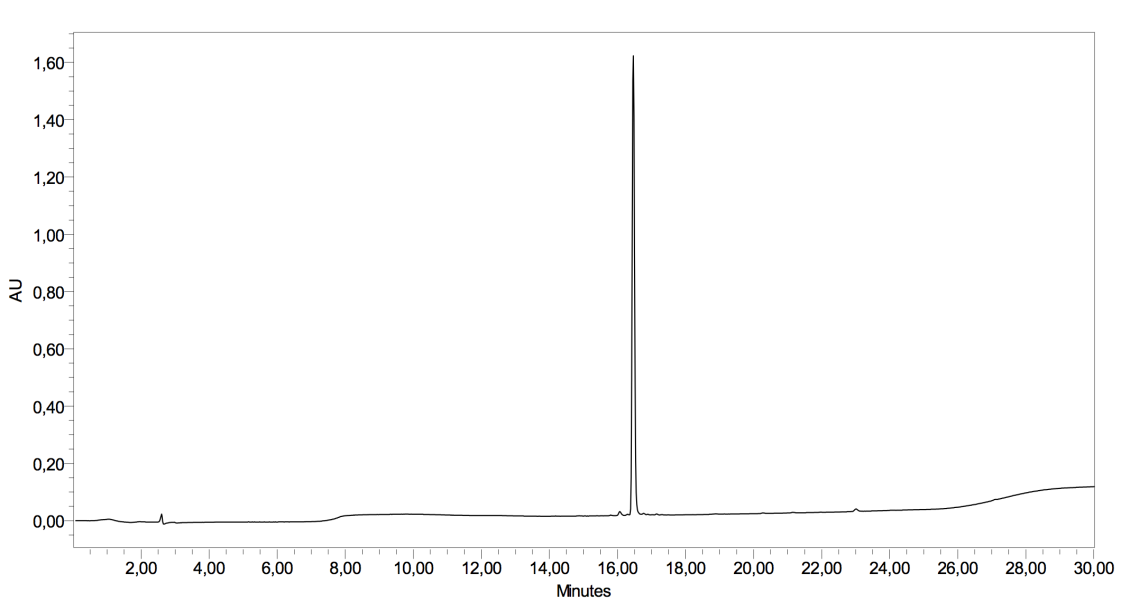


**Analytical HPLC chromatogram of 20**


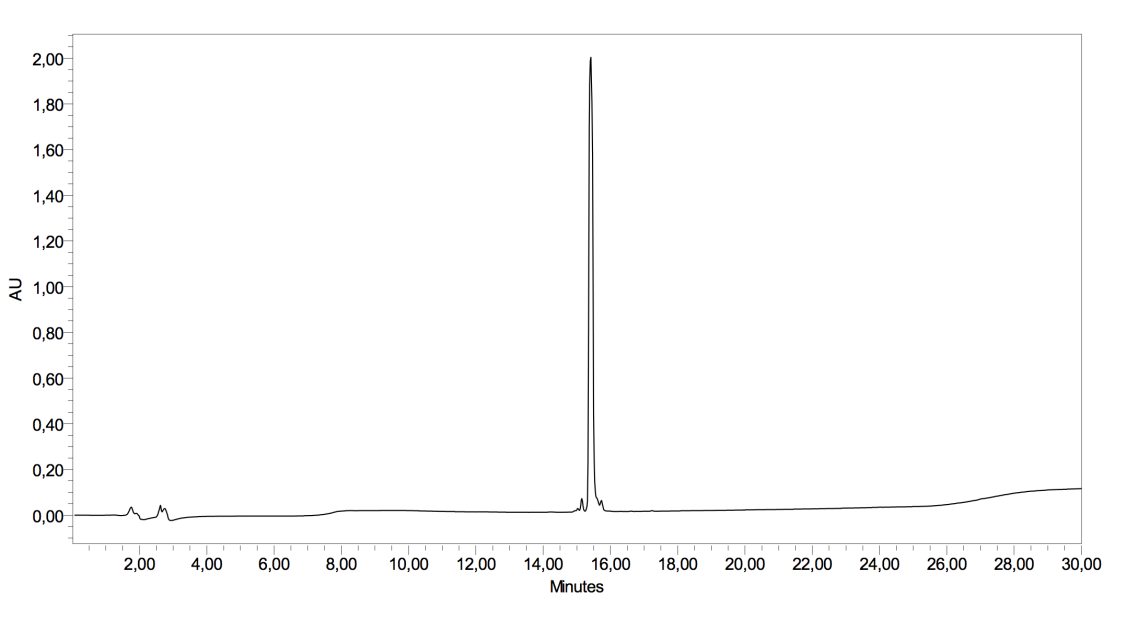


**Analytical HPLC chromatogram of 21**


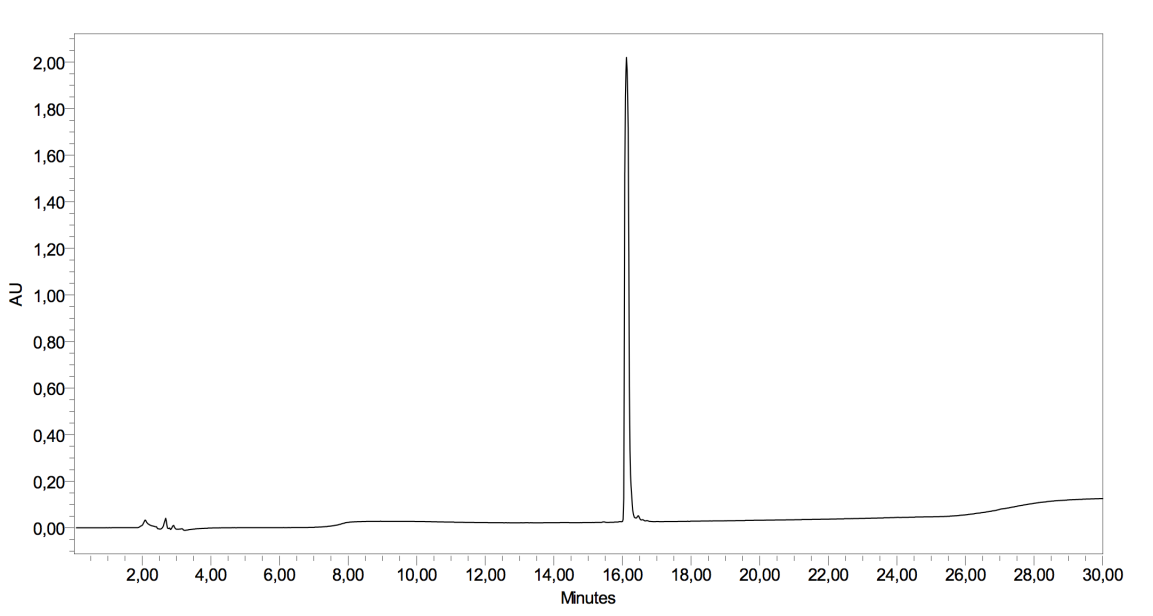


**Analytical HPLC chromatogram of 22**


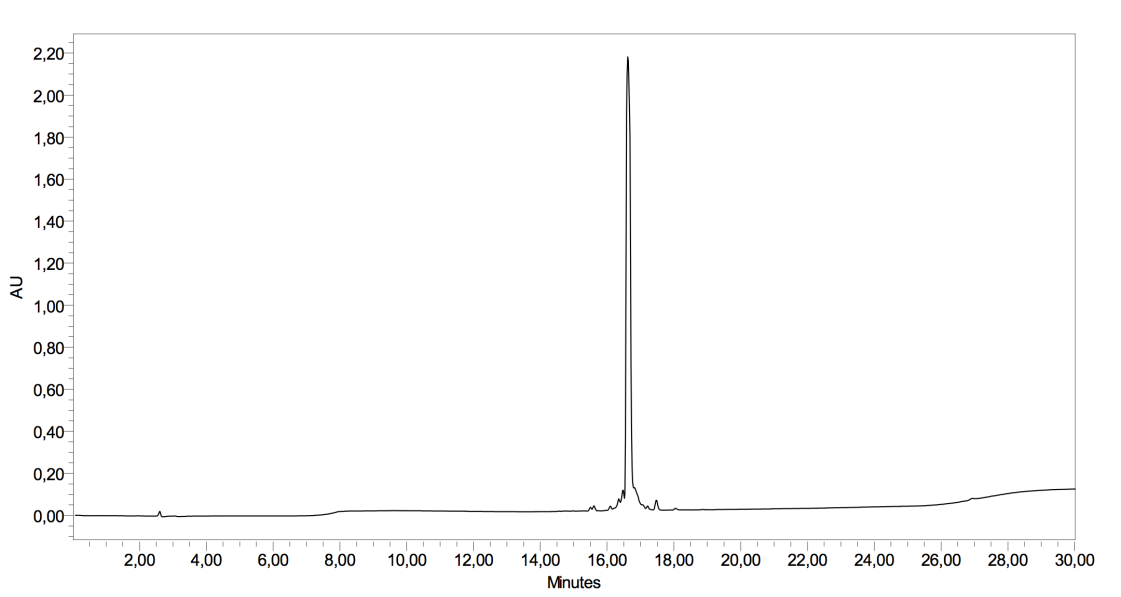


**Analytical HPLC chromatogram of 23**


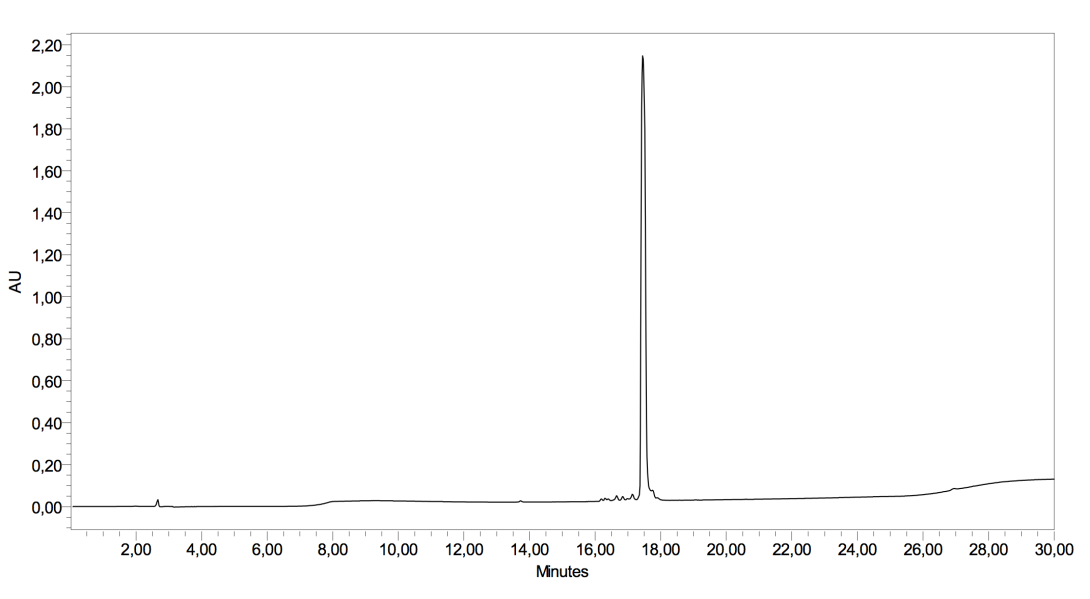


**Analytical HPLC chromatogram of 24**


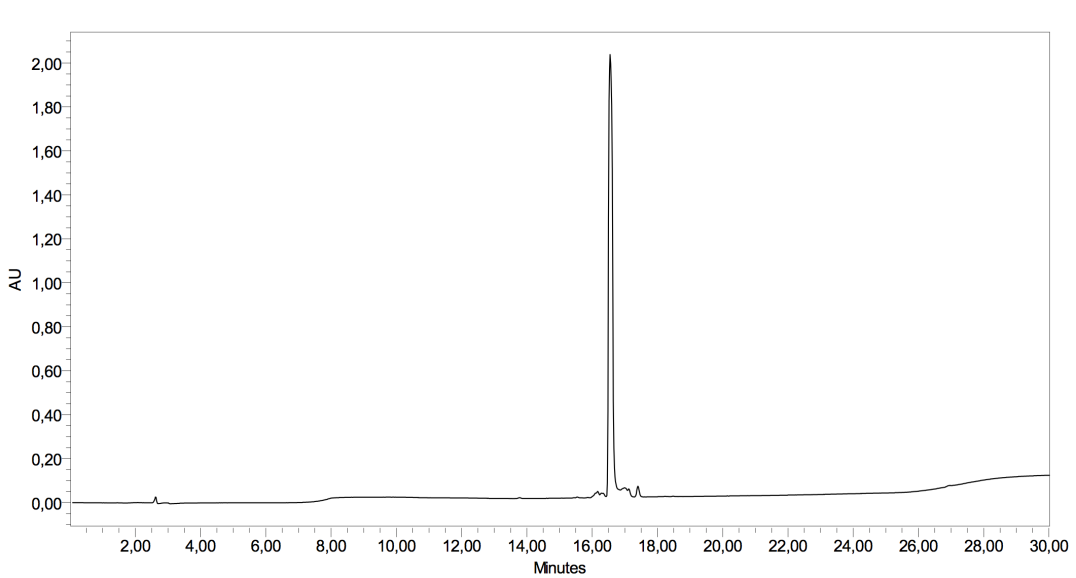


**Analytical HPLC chromatogram of 25**


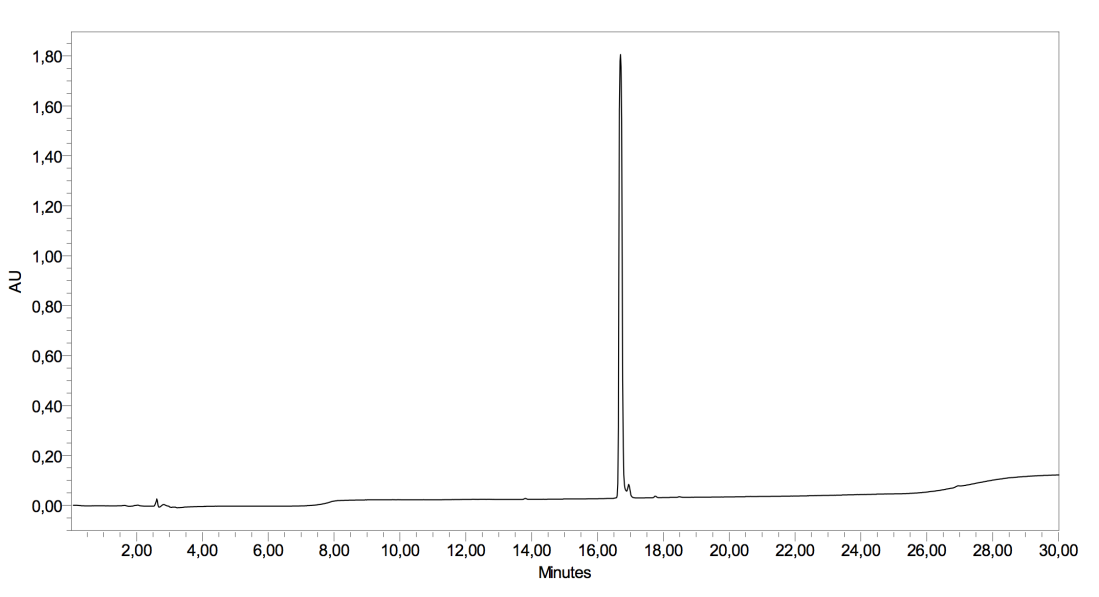


**Analytical HPLC chromatogram of 26**


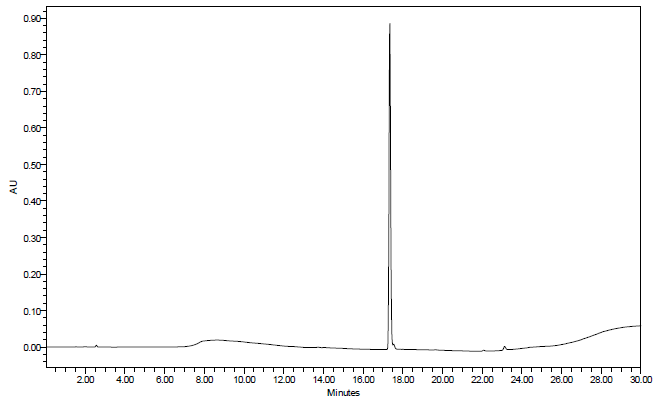


**Analytical HPLC chromatogram of 27**


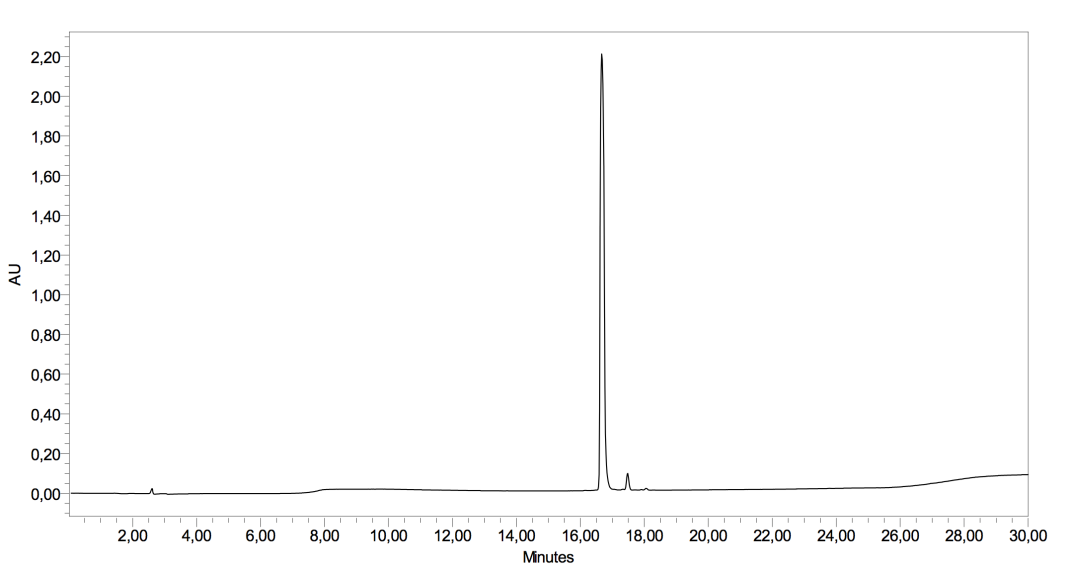


**Analytical HPLC chromatogram of 28**


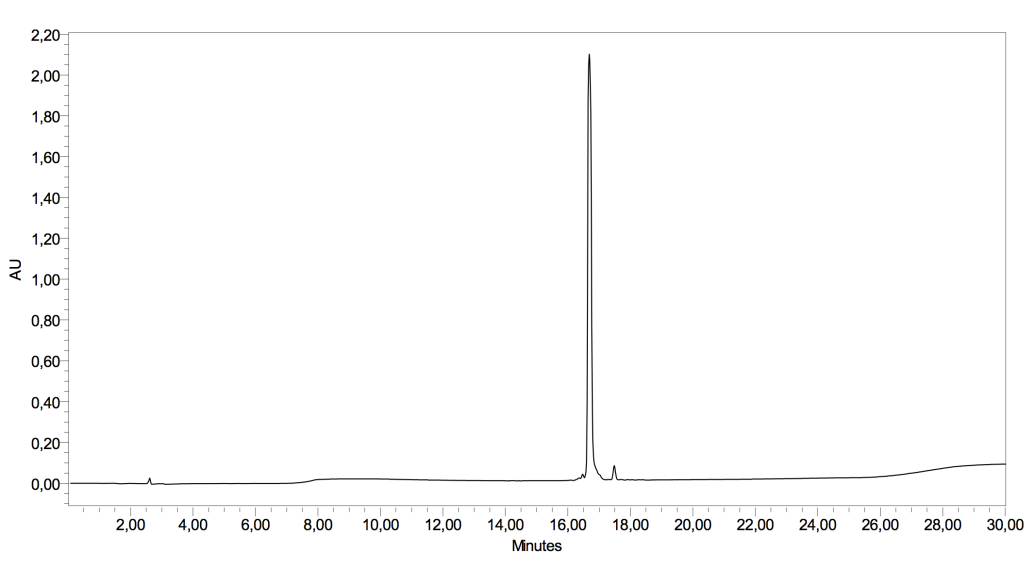


**Analytical HPLC chromatogram of 29**


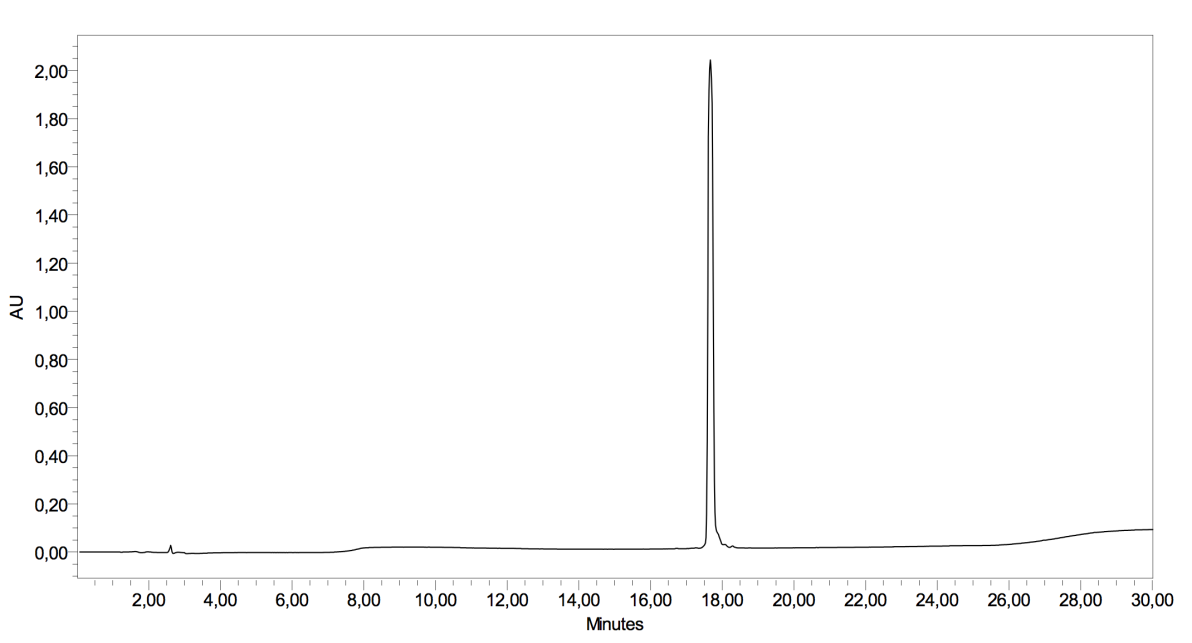


**S5: MIC distribution of MRSP, MSSP and *P. aeruginosa***

Supplementary Figure S5. MIC distribution (µg/ml) of MRSP (n=7), MSSP (n=50) *P. aeruginosa* (n=50).

**S6: High-throughput growth curves**

The effects of B1 on the growth of a reference MRSP strain (ST71, E104) were evaluated using BioScreen (Oy Growth Curves Ab LTd, Finland). A well characterized antimicrobial peptide, Nisin, was used as a positive control. Microbroth cultures of bacteria, with and without antimicrobial at MIC and sub-MIC concentrations, were distributed in a 100 well honeycomb micro-plate and incubated for 6 h at 37° C with continuous shaking. The OD600 of each micro-culture was measured and recorded every 15 min after a 5 second pause of shaking in automatic mode. The recorded ODs were plotted against time to prepare the growth curves. The assay was performed with technical and biological replicates, and one representative result was presented.


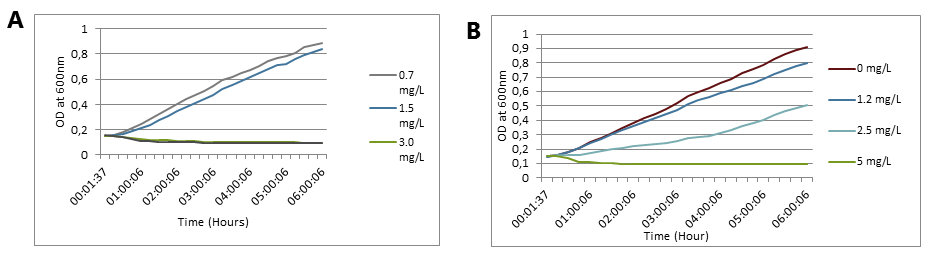


Supplementary Figure S6. Effects of B1 (a) and nisin (b) on growth of MRSP E104. Cells were grown up to 0.2 OD and then incubated with different concentrations of B1 or nisin for 6 h with a control (no antimicrobial). OD600nm was recorded every 15 min. In the right panel antimicrobial concentrations are listed, which follow the same order as the plotted growth curve lines, from top to bottom.

**Supplementary Table S8: Selectivity of 23 and 26 against *S. aureus* vs *S. pseudintermedius* strains**

| **Table S7: Selectivity of 23 and 26 against *S. aureus*** vs ***S. pseudintermedius*** strains | | |
| --- | --- | --- |
| **Bacteria** | **23** | **26** |
| ***S. aureus*** strains | | |
| 25054, 8 G6, dog, wound, 2009 | 64 | >64 |
| 27266, 16 G9, dog, skin, 2010 (also used in exp above) | 32-64 | 64 |
| 28264, 20 B1, dog, wound, 2011 | 64 | >64 |
| 30935, 24 B9, dog, joint, 2013 | 64 | >64 |
| 36968, 61 A9, dog, wound, 2016 | 64 | >64 |
| 37595, 65 D2, dog, joint, 2016 | >64 | >64 |
| 37708-2, 66 C6, dog, skin, 2016 | 32 | >64 |
| 38200, 68 E9, dog, skin, 2016 | 64 | >64 |
| 38565-1, 70 A5, dog, skin, 2017 | 64 | >64 |
| 38841, 70 G5, dog, urine, 2017 | 32 | 64 |
| ***S. pseudintermedius*** strains | | |
| 26071, 11 E5, dog, skin, 2009 | 8 | 4 |
| 26092-2, 11 E4, dog, skin, 2009 | 8 | 4 |
| 26959, 15 F8, dog, wound, 2010 | 4 | 4 |
| 27364, 17 A7, dog, wound, 2011 | 2 | 2 |
| 27382, 17 B8, dog, ear, 2011 | 4 | 2 |
| 31524, 54 C3, dog, ear, 2013 | 8 | 32 |
| 33228, 55 E2, dog, skin, 2014 | 2 | 2 |
| 35890, 59 B8, dog, ear, 2015 | 2 | 4 |
| 37526-1, 65 B4, dog, skin, 2016 | 1-2 | 4 |
| 37535-1, 65 B6, dog, skin, 2016 | 16 | 16 |
| 38637, 70 C3, dog, wound 2017 | 16 | 8 |
| 38820, 70 F9, dog, urine 2017 | 16 | 8 |
